# Flavonoid oligomers from Chinese dragon’s blood, the red resins of *Dracaena cochinchinensis*

**DOI:** 10.1007/s13659-012-0020-5

**Published:** 2012-04-11

**Authors:** Qing-An Zheng, Min Xu, Chong-Ren Yang, Dong Wang, Hai-Zhou Li, Hong-Tao Zhu, Ying-Jun Zhang

**Affiliations:** 1 State Key Laboratory of Phytochemistry and Plant Resources in West China, Kunming Institute of Botany, Chinese Academy of Sciences, Kunming, 650201 China; 2 Weihe Biotech Laboratory, Yuxi, 653100 China

**Keywords:** *Dracaena cochinchinensis*, flavonoid oligomers, cochinchinenins D-H

## Abstract

A detailed chemical investigation of the red resins from *Dracaena cochinchinensis* (Chinese dragon’s blood) yielded five new flavonoid oligomers, named cochinchinenins D-H (**1**–**5**), together with a known biflavonoid, cinnabarone (**6**), and a mixture of two known biflavonoids, socotrin-4′-ol (**7**) and homoisosocotrin-4′-ol (**8**). Of these new compounds, **1**–**3** were biflavonoids and **4** and **5** were triflavonoids. Their structures were determined on the basis of spectroscopic analysis. The isolated compounds were tested for cytotoxicity (Cdc25), antibacterial (PEPT) and antifungal (YNG) activities.

**Electronic Supplementary Material:**

Supplementary material is available for this article at 10.1007/s13659-012-0020-5 and is accessible for authorized users.

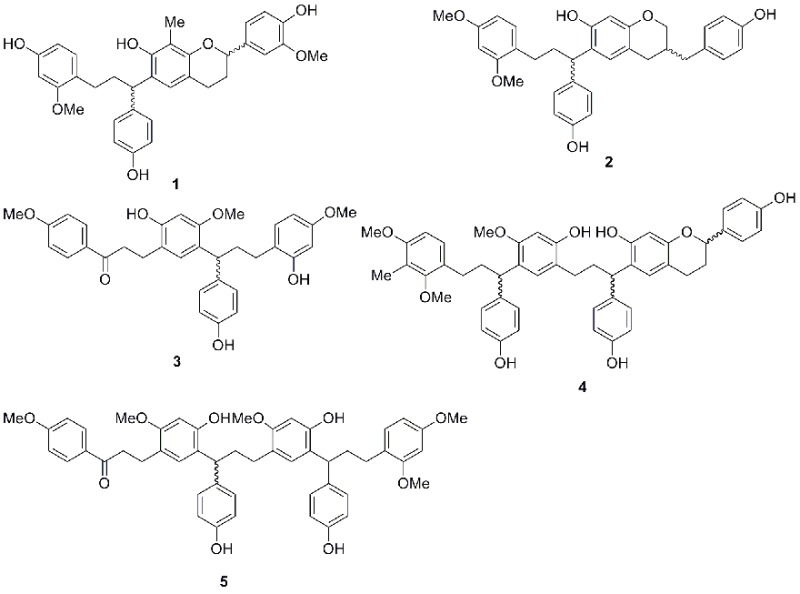

## Introduction

The resins excreted from stems of several disparate taxa, i.e. *Dracaena* (Agavaceae), *Croton* (Euphorbiaceae), *Pterocarpus* (Leguminosa) and *Daemonorops* (Palmae), have been used traditionally from ancient times as dragon's blood for the treatment of traumatic and visceral hemorrhages.[1] In China, dragon's blood has been imported and used traditionally as an important traditional Chinese medicinal herb for the treatment of traumatic and visceral hemorrhages since Tang dynasty.[2] Until 1970s, the red resin of *Dracaena cochinchinensis* S. C. Chen (Agavaceae) found in the southwest of China has been used widely as the substitute for the traditionally imported dragon's blood.[3] Chemical studies on the dragon's blood from the genus *Dracaena* (*D. draco*, *D. cinnabari* and *D. loureiri*) have revealed the occurrence of flavonoids, including flavan, flavone, chalcones, homoisoflavanes, and the oligomers of flavonoids.[4–16] Flavonoid oligomers, composing of one dihydrochalcone unit condensed with one or more chalcone, flavane or homoisoflavane units,[5–7] constituted the major identified components of dragon's blood from the genus *Dracaena*. Furthermore, a series of publications reported the natural product diversity of original plants of dragon's blood from the genus *Dracaena*.[17–28] These natural products from Dragon's blood and its original plants showed cytotoxic, antibacterial, and antioxidant activities.[15,29,30]

*To whom correspondence should be addressed. E-mail: zhangyj@mail.kib.ac.cn (Y.J. Zhang); xumin@mail.kib.ac.cn (M. Xu).

**Figure 1**. Compounds isolated from Chinese dragon's blood, the red resins of *Dracaena cochinchinensis*

*D. cochinchinensis* S. C. Chen is an evergreen tree or shrub native to the tropical region of southwestern China, Myanmar, and Laos. The red resin excreted from the stems was originally used by the Dai people living in the southern part of Yunnan province, China, for the treatment of pain and hemorrhages. From 1970s, it has been used widely as the main source of dragon's blood, called Long-Xue-Jie (Chinese dragon's blood), for the treatment of traumatic and visceral hemorrhages.[3] Previous studies showed that the resin contains mainly of phenolic compounds, as well as several steroids and aliphatic acids.[30–38]

In our continuing studies on Chinese dragon's blood, five new flavonoid oligomers namely cochinchinenins D–H (**1**–**5**) were further isolated and identified from the red resin of *D. cochinchinesis*, in addition to the known biflavonoid, cinnabarone (**6**), and a mixture of two known biflavonoids, socotrin-4'-ol (**7**) and homoisosocotrin-4'-ol (**8**). Their structures were elucidated on the basis of detailed spectroscopic analysis. The cytotoxicity (CDC25), antibacterial (PEPT) and antifungal (YNG) activities of the isolated compounds **1**–**8** were also evaluated.

## Results and Discussion

The $CHCl_3$ extract of the red resin from *D. cochinchinensis* was subjected to a silica gel column and eluted with $CHCl_3$/MeOH. Six fractions were yielded. Fractions 1–4 contained mainly simple flavonoids.[30] Further detailed study on fractions 5 and 6 led to the identification of eight flavonoid oligomers (**1**–**8**). Of these, **6** was the known biflavonoid, cinnabarone,[8] while **7** and **8** were obtained as a mixture of two known biflavonoids, socotrin-4'-ol (**7**)[7] and homoisosocotrin-4'-ol (**8**).[7] The known compounds (**6**–**8**) were elucidated by comparison of their spectroscopic data with the reported literature values.

Compound **1** was obtained as a brown amorphous powder and had a molecular formula $C_{33}H_{34}O_7$, as deduced by the positive ion HRESIMS (*m/z* 542.2261 $[M]^+$) and the $^{13}C$ NMR (Table 1), implying 17 degrees of unsaturation. All 33 carbon resonances were well resolved in the $^{13}C$ NMR spectrum (Table 1) and further classified by DEPT and HSQC experiments as 13 quaternary aromatic carbons with seven bearing oxygen, 13 methines with 11 aromatic carbons, four aliphatic methylenes, one shielded methyl ($\delta$ 9.1) and two methoxys ($\delta$ 55.6 and 56.4). The aromatic region of the $^1H$ NMR spectrum of **1** displayed two two-proton doublets [$\delta$ 7.10 and 6.70 (each 2H, $J$ = 8.6 Hz)] arising from a 1,4-disubstituted benzene ring. In addition, two sets of ABX coupled signals were ascribable to two 1,2,4-trisubstituted aromatic rings, and one-proton singlet ($\delta$ 6.75) was due to a pentasubstituted aromatic ring. Moreover, 19 aliphatic protons arising from four $CH_2$, two CH, one $CH_3$ ($\delta$ 2.03, s) and two $OCH_3$ ($\delta$ 3.75, 3.83, each s) were observed. These aforementioned $^1H$ NMR data of **1** were similar to those of socotrin-4'-ol (**7**),[3] except for the substitutions of rings A and B, and the appearance of two additional methyl groups in **1**. One of the methyls was a methoxy at an aromatic ring ($\delta$ 56.4), while the other ($\delta$ 9.1) was linked to an aromatic ring directly.

Detailed analysis of the $^1H$-$^1H$ COSY, HSQC, and HMBC spectra (Figure 1) revealed that **1** was a biflavonoid composed of a deoxotetrahydrochalcone unit and a flavan unit. Firstly, HSQC correlations led to the assignments of the protons to their corresponding carbons. The long-range correlations observed in the HMBC spectrum enabled the assignments of each proton and carbon signal of the four aromatic rings and the connection between these substructures, as shown in **1** (Figure 2). In the HMBC spectrum, the correlation of oxygen-bearing methine proton at $\delta$ 4.91 (H-2) with aromatic carbons at $\delta$ 135.5 (C-1'), 110.8 (C-2') and 119.7 (C-6') indicated the 1,3,4-trisubstituted aromatic ring as B ring. The aromatic ring was further confirmed to be 3'-methoxy-4'-hydroxy substitution by HMBC cross peaks of H-2' with C-6'/C-4', H-5' with C-3'/C-1', H-6' with C-2'/C-4', as well as of the methoxy protons at $\delta$ 3.83 (s) with C-3'. Correlations of the methylene protons at $\delta$ 2.48 (2H, H-$\beta$) were observed with aromatic carbons at $\delta$ 160.0 (C-2'') and 131.1(C-6''). The corresponding protons at $\delta$ 6.85 (d, $J$ = 8.0 Hz), 6.29 (dd, $J$ = 8.0, 2.3 Hz) and 6.39 (d, $J$ = 2.3 Hz) indicated that D-ring was a 1,2,4-trisubstituted aromatic ring, on which a hydroxy and a methoxy group were linked at C-4'' and C-2'', respectively. This was confirmed by the cross peak from the methoxy proton at $\delta$ 3.75 and C-2'' ($\delta$ 160.0). Moreover, HMBC correlations of the CH proton at $\delta$ 4.24 with aromatic carbons at $\delta$ 130.5 (C-2''' and 6'''), 152.5 (C-7) and 126.0 (C-5) revealed that the E ring was a 1,4-disubsitututed aromatic ring with a hydroxyl group at C-4''', and the A ring was a pentasubstituted aromatic ring with a hydroxyl group and methyl at C-7 and C-8, respectively. This assignment was confirmed by the HMBC correlations of H-5 with $\delta$ 152.5 (C-7)/153.1 (C-8a), and $CH_3$ ($\delta$ 2.03) with $\delta$ 112.6 (C-8)/152.5 (C-7)/153.1 (C-8a). Therefore, the structure of cochinchinenin D was determined as shown in Figure 1.

**Figure 2**. $^1H$-$^1H$ COSY and selected HMBC correlations of **1**

The molecular formula of compound **2** was assigned as $C_{33}H_{34}O_6$ on the basis of its $^{13}C$ NMR data (Table 1) and positive HRESIMS, which was 14 Da heavier than that of homoisosocotrin-4'-ol (**8**).[3] The $^1H$ and $^{13}C$ NMR data of **2** were very similar to those of **8**, except for an additional oxygen-bearing methyl group, indicating that **2** was a methyl ether of **8**. The position of the additional methoxy group in **2** was revealed to be at C-4'' by the HMBC correlation of a methoxy group at $\delta$ 3.53 with an oxygen-bearing aromatic carbon at $\delta$ 159.5 (C-4''). Other HMBC and $^1H$-$^1H$ COSY correlations (Electronic Supplementary Material) confirmed the structure of **2**. Accordingly, compound **2** was deduced to be the 4''-methyl ether of homoisosocotrin-4'-ol, and named cochinchinenin E.

**Table 1. NMR spectroscopic data [100 ($^{13}C$) and 400 ($^{1}H$) MHz, $CD_3OD$] for compounds 1–3**

| position | 1 | | 2 | | 3 | |
|---|---|---|---|---|---|---|
| | $\delta_C$ | $\delta_H$ ($J$ in Hz) | $\delta_C$ | $\delta_H$ ($J$ in Hz) | $\delta_C$ | $\delta_H$ ($J$ in Hz) |
| 1 | | | | | 123.2 (C) | |
| 2 | 79.2 (CH) | 4.91, m | 70.7 ($CH_2$) | 4.03, m; 3.82, m | 155.9 (C) | |
| 3 | 32.0 ($CH_2$) | 2.46, m; 2.11, m | 30.0 (CH) | 2.11, m | 99.7 (CH) | 6.36, s |
| 4 | 26.5 ($CH_2$) | 2.86, m; 2.66, m | 31.4 ($CH_2$) | 2.86, m; 2.66, m | 157.5 (C) | |
| 4a | 113.4 (C) | | 113.3 (C) | | | |
| 5 | 126.0 (CH) | 6.75, s | 129.6 (CH) | 6.56, s | 120.7 (C) | |
| 6 | 126.6 (C) | | 125.9 (C) | | 130.1 (CH) | 6.90, s |
| 7 | 152.5 (C) | | 154.6 (C) | | | |
| 8 | 112.6 (C) | | 103.3 (CH) | 6.02, s | | |
| 8a | 153.1 (C) | | 155.2 | | | |
| 9 | | | 37.9 ($CH_2$) | 2.30, m | | |
| 1' | 135.5 (C) | | 131.7 (C) | | 138.3 (C) | |
| 2' | 110.8 (CH) | 7.01, d (1.8) | 129.6 (CH) | 6.79, d (8.6) | 131.7 (CH) | 7.85, d (6.8) |
| 3' | 148.9 (C) | | 116.0 (CH) | 6.58, d (8.6) | 115.7 (CH) | 6.80, d (6.8) |
| 4' | 146.9 (C) | | 157.3 (C) | | 165.2 (C) | |
| 5' | 116.0 (CH) | 6.83, d (8.0) | 116.0 (CH) | 6.58, d (8.6) | 115.7 (CH) | 6.80, d (6.8) |
| 6' | 119.7 (CH) | 6.92, dd (8.0, 1.8) | 129.6 (CH) | 6.79, d (8.6) | 131.7 (CH) | 7.85, d (6.8) |
| 1" | 123.3 (C) | | 131.6 (C) | | 124.8 (C) | |
| 2" | 160.0 (C) | | 158.7 (C) | | 159.7 (C) | |
| 3" | 99.8 (CH) | 6.39, d (2.3) | 99.5 (CH) | 6.35, d (2.3) | 99.8 (CH) | 6.39, br. s |
| 4" | 157.6 (C) | | 159.5 (C) | | 157.5 (C) | |
| 5" | 107.6 (CH) | 6.29, dd (8.0,2.3) | 107.3 (CH) | 6.25, dd (2.3, 8.1) | 107.5 (CH) | 6.28, dd (7.9, 2.4) |
| 6" | 131.1 (CH) | 6.85, d (8.0) | 130.9 (CH) | 6.80, d (8.1) | 131.1 (CH) | 6.83, d (7.9) |
| $\alpha$ | 37.8 ($CH_2$) | 2.14, m | 37.0 ($CH_2$) | 2.14, m | 40.0 ($CH_2$) | 3.11, m |
| $\beta$ | 30.0 ($CH_2$) | 2.48, m | 29.4 ($CH_2$) | 2.48, m | 27.3 ($CH_2$) | 2.88, m |
| CO | | | | | 202.2 (C) | |
| CH | 44.1 (CH) | 4.24, t (7.6) | 43.0 (CH) | 4.24, t (7.6) | 43.1 (CH) | 4.17, t (7.9) |
| $\alpha'$ | | | | | 37.2 ($CH_2$) | 2.06, m |
| $\beta'$ | | | | | 29.5 ($CH_2$) | 2.38, m |
| 1''' | 138.7 (CH) | | 139.1 (C) | | 130.1 (C) | |
| 2''',6''' | 130.5 (CH) | 7.10, d (8.6) | 129.8 (CH) | 6.97, d (8.6) | 130.0 (CH) | 7.06, d (7.0) |
| 3''',5''' | 116.2 (CH) | 6.70, d (8.6) | 114.2 (CH) | 6.55, d (8.6) | 115.7 (CH) | 6.66, d (7.0) |
| 4''' | 156.5 (C) | | 157.3 (C) | | 155.1 (C) | |
| $CH_3$ | 9.1 ($CH_3$) | 2.03, s | | | | |
| $OCH_3$ | 55.6 ($CH_3$) | 3.75, s | 55.4 ($CH_3$) | 3.54, s | 55.6 ($CH_3$) | 3.75, s |
| $OCH_3$ | 56.4 ($CH_3$) | 3.83, s | 55.4 ($CH_3$) | 3.53, s | 55.7 ($CH_3$) | 3.75, s |
| $OCH_3$ | | | | | 55.9 ($CH_3$) | 3.81, s |

Compound **3** was a brown amorphous powder and showed a molecular ion peak in its positive ion HRESIMS corresponding to the molecular formula $C_{33}H_{34}O_7$, which was 14 mass units more than that of cinnabarone (**6**).[8] The $^{1}H$ and $^{13}C$ NMR spectra (Table 1) of **3** were closely related to those of **6**,[8] except for an additional oxidized methyl group, indicating that **3** was a methyl ether of **6**. Careful comparison of the NMR data of **3** with those of **6** showed that the signal assigned to C-4' was deshielded to $\delta$ 165.2 (from $\delta$ 163.6 in **6**), while the other signals were quite similar with each other. These revealed that the C-4' of compound **3** was substituted by a methoxyl group relative to **6**. The HMBC and $^{1}H$-$^{1}H$ COSY correlations (Electronic Supplementary Material) further confirmed the structure of **3.** Thus, cochinchinenin F was characterized as the 4'-methyl ether of cinnabarone, as shown in Figure 1.

The molecular formula of compound **4** was deduced as $C_{49}H_{50}O_9$, on the basis of its negative ion HRFABMS and its NMR spectroscopic data. The $^{13}C$ NMR spectrum of **4** showed the presence of 49 carbon signals, including 18 quaternary with nine oxygen-bearing ones, 18 tertiary $sp^2$ carbons, ascribable to six aromatic rings, as well as 13 aliphatic carbons comprising six methylenes [$\delta$ 31.6 ($CH_2$), 26.2 ($CH_2$), 37.3 ($CH_2$ × 2) and 29.7 ($CH_2$ × 2)], three methines [$\delta$ 78.8 (CH) and 43.2 (CH × 2)], three methoxys [$\delta$ 55.6 ($OCH_3$ × 2 ) and 55.8 ($OCH_3$)] and one methyl [$\delta$ 9.1 ($CH_3$)]. Among them, the shielded methyl carbon at $\delta$ 9.1 suggested it was connected directly to a phenyl group. The $^{1}H$ NMR spectrum displayed 27 aliphatic protons including three singlets at $\delta$ 3.72 (6H), 3.68 (3H), and 1.90 (3H) assignable to three methoxys and one methyl, two multiplets at $\delta$ 4.65 (1H) and 4.20 (2H) and 18 aromatic protons. These data indicated that **4** contained one flavane and two deoxotetrahydrochalcone units. Comparison of its NMR signals with those of damalchawin (**9**) suggested that both compounds had similar skeletons with different substitutions.[5] Compound **4** had one methoxy, one methyl and one hydroxy group more than **9**. On the basis of detailed analysis of the $^{1}H$-$^{1}H$ COSY experiment (Electronic Supplementary Material), the aromatic signals were distributed into three 1,4-disubstituted (three $A_2B_2$ spin systems, B, E and G rings), two 1,2,4,5-tetrasubstituted (four singlets, A and D rings) and one 1,2,3,4-tetrasubstituted (an AB spin system at $\delta$ 6.24 and 6.72, F ring) aromatic rings. The long-range connectivity observed in the HMBC spectrum enabled the connection between these aromatic substructures as shown in structure **4** (Electronic Supplementary Material). The two CH protons showed correlations to two $A_2B_2$ spin systems (E, G rings), respectively. The H-$\beta$ and H-$\beta'$ ($\delta$ 2.42) signals showed correlations to one 1,2,4,5-tetrasubstituted aromatic ring (D ring) and an AB spin system (F ring). In the EIMS of **4** (Figure 3), diagnostic fragments were detected at $m/z$ 286 (**4a**), 256 (**4b**), 242 (**4c**) [splitting into three monomers], 137 [benzyl cleavage], and a base peak at $m/z$ 120 [benzyl cleavage], which clarified the subunits of the triflavonoid (Figure 2) and further confirmed the structure of cochinchinenin G as shown in Figure 1.

**Figure 3.** Characteristic EIMS fragments of compound **4**

Cochinchinenin H (**5**), a brown amorphous powder, possessed a molecular formula $C_{50}H_{52}O_{10}$, as deduced from the negative ion HRFABMS and the NMR spectroscopic data, implying 25 unsaturated degrees. The $^{13}$C NMR spectrum of **5** displayed the presence of 50 carbon signals, including one carbonyl ($\delta$ 202.0), 17 quaternary with nine bearing oxygen, and 19 tertiary $sp^2$ carbons. These data suggested the existence of six benzene rings, in addition to 13 $sp^3$ carbons. The $^1$H NMR spectrum showed 19 aromatic and 29 aliphatic protons. These NMR data suggested compound **5** was a triflavonoid composing of one dihydrochalcone and two deoxotetrahydrochalcone units. From a detailed analysis of the $^1$H-$^1$H COSY and HSQC correlations (Electronic Supplementary Material), the aromatic signals were arranged to three 1,4-disubstituted (three $A_2B_2$ spin system, B, D, and F rings), two 1,2,4,5-tetrasubstituted (four singlets, A and C rings) and one 1,2,4-trisubstituted (an ABX spin system, E ring) aromatic rings, whereas the aliphatic protons were ascribable to five methoxys [$\delta$ 3.72 (3H), 3.68 (9H), 3.64 (3H)], two methines [$\delta$ 4.18 (2H, m)], and six methylenes. In the HMBC spectrum of **5**, correlations of the two CH protons ($\delta$ 4.18, m, 2H) with two $A_2B_2$ spin systems (D and F rings) and two 1,2,4,5-tetrasubstituted (A and C rings) were observed (Electronic Supplementary Material). The HMBC correlations of H-$\beta$ ($\delta$ 2.86, m) with the A ring carbons [$\delta$ 123.2 (C-1), C-2 (157.3), 131.2 (C-6)] and the carbonyl at $\delta$ 202.0 further confirmed the connection of dihydrochalcone unit and one deoxotetrahydrochalcone unit. Moreover, the HMBC correlations of H-$\beta'$ ($\delta$ 2.38, m) with $\delta$ 157.5 (C-2'') and 131.2 (C-6'') from the C ring, and H-$\beta''$ ($\delta$ 2.38, m) with $\delta$ 158.9 (C-2''''') and 132.0 (C-6''''') of the E ring indicated the connection of an additional deoxotetrahydrochalcone unit. The oxidized methyl groups $\delta$ 3.72, 3.68 and 3.64 showed cross peaks with carbon atoms $\delta$ 165.1 (C-4'), 157.3 (C-2), 157.5 (C-2''), 159.7 (C-4'''''), 158.9 (C-2'''''), respectively, indicating the position of these groups. Thus, the structure of cochinchinenin H (**5**) was clarified as shown in Figure 1.

Flavonoids have been found to be characteristic components in dragon's blood from the genus *Dracaena*. Though some flavans, flavones, chalcones and homoisoflavanes were reported from the red resin, only 12 biflavonoids (cinnabarone, 2'-methoxy-socotrin-5'-ol, socotrin-4'-ol, homoisosocotrin-4'-ol, cochinchinenin, cochinchinenenes A–D, (2*R*)-8-methyl-socotrin-4'-ol, cochinchinenins B and C) and one triflavonoid (damalachawin) were identified as flavonoid oligomers.[5,7,30,32] The present study led to the further identification of three new biflavonoids (**1**–**3**) and two new triflavonoids (**4** and **5**) from the red resin of *D. cochinchinensis*.

**Table 2. NMR spectroscopic data [100 ($^{13}$C) and 400 ($^1$H) MHz, $CD_3OD$] for compounds 4 and 5**

| | **4** | | **5** | |
|---|---|---|---|---|
| pos. | $\delta_C$ | $\delta_H$ ($J$ in Hz) | $\delta_C$ | $\delta_H$ ($J$ in Hz) |
| 1 | | | 123.2 (C) | |
| 2 | 78.8 (CH) | 4.65, m | 157.3 (C) | |
| 3 | 26.2 ($CH_2$) | 2.42, m | 99.7 (CH) | 6.34, s |
| 4 | 31.6 ($CH_2$) | 2.70, m | 155.9 (C) | |
| 4a | 113.5 (C) | | | |
| 5 | 129.6 (CH) | 6.77, s | 120.7 (C) | |
| 6 | 126.6 (C) | | 131.2 (CH) | 6.80, s |
| 7 | 156.0 (C) | | | |
| 8 | 107.5 (CH) | 6.68, s | | |
| 8a | 154.6 (C) | | | |
| 1' | 134.8 (C) | | 138.4 (C) | |
| 2',6' | 128.3 (CH) | 7.07, d (8.6) | 131.7 (CH) | 7.80, d (8.5) |
| 3',5' | 114.4 (CH) | 6.68, d (8.6) | 115.7 (CH) | 6.80, d (8.5) |
| 4' | 157.3 (C) | | 165.1 (C) | |
| CH | 43.2 (CH) | 4.20, m | 43.3 (CH) | 4.18, m |
| $\alpha$ | 37.3 ($CH_2$) | 2.03, m | 40.1 ($CH_2$) | 3.07, m |
| $\beta$ | 29.7 ($CH_2$) | 2.42, m | 27.2 ($CH_2$) | 2.86, m |
| CO | | | 202.0 (C) | |
| 1'' | 122.6 (C) | | 124.8 (C) | |
| 2'' | 152.7 (C) | | 157.5 (C) | |
| 3'' | 99.7 (CH) | 6.35, s | 99.7 (CH) | 6.33, s |
| 4'' | 157.5 (C) | | 149.6 (C) | |
| 5'' | 124.5 (C) | | 120.7 (C) | |
| 6'' | 130.1 (CH) | 6.68, s | 131.2 (CH) | 6.76, s |
| 1''' | 139.6 (C) | | 139.4 (C) | |
| 2''' | 130.2 (CH) | 7.24, d (8.5) | 130.1 (CH) | 7.06, d (8.6) |
| 3''' | 116.0 (CH) | 6.78, d (8.5) | 114.3 (CH) | 6.68, d (8.6) |
| 4''' | 157.3 (C) | | 155.2 (C) | |
| 5''' | 116.0 (CH) | 6.78, d (8.5) | 114.3 (CH) | 6.68, d (8.6) |
| 6''' | 130.2 (CH) | 7.24, d (8.5) | 130.1 (CH) | 7.06, d (8.6) |
| CH' | 43.2 (CH) | 4.20, m | 43.2 (CH) | 4.18, m |
| $\alpha'$ | 37.3 ($CH_2$) | 2.03, m | 37.4 ($CH_2$) | 2.06, m |
| $\beta'$ | 29.7 ($CH_2$) | 2.42, m | 29.7 ($CH_2$) | 2.38, m |
| $\alpha''$ | | | 37.3 ($CH_2$) | 2.06, m |
| $\beta''$ | | | 29.7 ($CH_2$) | 2.38, m |
| 1'''' | 123.3 (C) | | 138.4 (C) | |
| 2'''' | 159.7 (C) | | 130.1 (CH) | 7.06, d (8.6) |
| 3'''' | 126.1 (C) | | 114.7 (CH) | 6.68, d (8.6) |
| 4'''' | 158.9 (C) | | 156.6 (C) | |
| 5'''' | 99.8 (CH) | 6.24, d (8.5) | 114.7 (CH) | 6.68, d (8.6) |
| 6'''' | 131.1 (CH) | 6.72, d (8.5) | 130.1 (CH) | 7.06, d (8.6) |
| 1''''' | 138.5 (C) | | 125.0 (C) | |
| 2''''' | 130.2 (CH) | 7.24, d (8.5) | 158.9 (C) | |
| 3''''' | 116.0 (CH) | 6.78, d (8.5) | 99.8 (CH) | 6.33, s |
| 4''''' | 157.8 (C) | | 159.7 (C) | |
| 5''''' | 116.0 (CH) | 6.78, d (8.5) | 107.5 (CH) | 6.28, d (8.5) |
| 6''''' | 130.2 (CH) | 7.24, d (8.5) | 132.0 (CH) | 6.76, d (8.5) |
| $CH_3$ | 9.1 ($CH_3$) | 1.90, s | | |
| $OCH_3$ | 55.6 ($CH_3$) | 3.72, s | 55.6 ($CH_3$) | 3.68, s |
| $OCH_3$ | 55.6 ($CH_3$) | 3.72, s | 55.7 ($CH_3$) | 3.72, s |
| $OCH_3$ | 55.8 ($CH_3$) | 3.68, s | 55.8 ($CH_3$) | 3.64, s |

## Experimental Section

**General Experimental Procedures**. NMR spectra were run on Bruker AV-400 (for $^1$H and $^{13}$C NMR) and DRX-500 (for 2D NMR) instruments with TMS as internal standard; Mass spectra were recorded on a VG Auto Spec-3000 or API QSTAR Pulsar-I spectrometers. UV spectra were obtained on a Shimadzu double-beam 210A spectrophotometer. Silica gel (200–300 mesh and 10–40 $\mu$m), RP-18 (40–63 $\mu$m) and Sephadex LH-20 were used for column chromatography.

**Plant Material.** The red resin of *D. cochinchinensis* was purchased from Weihe Pharmaceutical Company (Yuxi,

Yunnan, China). A sample was deposited in our laboratory. Identification of the extract was supported by an HPLC comparison with an authentic sample, which was Long-Xue-Jie (Chinese dragon's blood, No. 020624) provided by Xishuangbanna Botanical Garden, Chinese Academy of Sciences. A voucher specimen of *D. cochinchinensis* (KUN 0238050) is deposited in State Key Laboratory of Phytochemistry and Plant Resources in west China, Kunming Institute of Botany, Chinese Academy of Sciences.

**Extraction and Isolation.** The red resin (1.0 Kg) of *D. cochinchinensis* was extracted with $CHCl_3$, EtOAc, and MeOH, successively. The $CHCl_3$ extract (90 g) was subjected to silica gel CC and eluted with the following gradient: $CHCl_3$, $CHCl_3$/MeOH (20:1, 10:1, 10:2) and finally MeOH, to give 6 fractions (Fr. 1–6). Fr. 5 (10.0 g) and Fr. 6 (5.0 g) were subjected separately to repeated CC on silica gel ($CHCl_3$/MeOH, 20:1–4:1) and Sephadex LH-20 (MeOH) to yield **1** (30 mg), **2** (32 mg), **3** (7 mg), **6** (cinnabarone, 15 mg), and a mixture (15 mg) of **7** and **8** from Fr. 5, and **4** (100 mg) and **5** (20 mg) from Fr. 6, respectively.

**Cochinchinenin D (1):** brown amorphous powder; $[\alpha]_D^{17}$ +48.8 (*c* 0.20, MeOH); UV (MeOH) $\lambda_{max}$ nm (log $\varepsilon$): 206 (2.48), 280 (2.21); $^1$H and $^{13}$C NMR: see Table 1; EIMS *m/z* 542 $[M]^+$; HRESIMS (Positive ion mode) *m/z* 542.2261 $[M]^+$ (calcd for $C_{33}H_{34}O_7$, 542.2304).

**Cochinchinenin E (2):** brown amorphous powder; $[\alpha]_D^{17}$ +33.7 (*c* 0.30, MeOH). UV (MeOH) $\lambda_{max}$ nm (log $\varepsilon$): 207 (2.69), 285 (2.48); $^1$H and $^{13}$C NMR: see Table 1; EIMS *m/z* 526 $[M]^+$ (85), 375 (100), 361 (70), 270 (45), 151 (47), 137 (55), 107 (74), 77 (11); HRESIMS (Positive ion mode) *m/z* 526.2346 $[M]^+$ (calcd for $C_{33}H_{34}O_6$, 526.2355).

**Cochinchinenin F (3):** brown amorphous powder; $[\alpha]_D^{17}$ +8.0 (*c* 0.23, MeOH); UV (MeOH) $\lambda_{max}$ nm (log $\varepsilon$): 206 (2.60), 280 (2.58); $^1$H and $^{13}$C NMR: see Table 1; EIMS *m/z* 542 $[M]^+$; HRESIMS (positive ion mode) *m/z* 542.2303 $[M]^+$ (calcd for $C_{33}H_{34}O_7$, 542.2304).

**Cochinchinenin G (4):** brown amorphous powder; UV (MeOH) $\lambda_{max}$ nm (log $\varepsilon$): 207 (2.69), 285 (2.21); $^1$H and $^{13}$C NMR: see Table 2; FABMS (negative ion mode) *m/z* 781 $[M-H]^-$; HRFABMS (negative ion mode) *m/z* 781.3367 $[M-H]^-$ (calcd for $C_{49}H_{49}O_9$, 781.3376).

**Cochinchinenin H (5):** brown amorphous powder; $[\alpha]_D^{17}$ +5.1 (*c* 0.23, MeOH). UV (MeOH) $\lambda_{max}$ nm (log $\varepsilon$): 207 (2.69), 285 (2.58); $^1$H and $^{13}$C NMR: see Table 2; FABMS (negative ion mode) *m/z* 811 $[M-H]^-$; HRFABMS (negative mode) *m/z* 811.3450 $[M-H]^-$ (calcd for $C_{50}H_{51}O_{10}$, 811.3482).

**Cytotoxicity (Cdc25) Assay.** The cytotoxicity activity of the pure compounds against Cdc25 was determined using the method described previously.[39]

**Antibacterial (PEPT) Assay.** The assay employed is a microtiterplate adaptation of a phosphate detection method described previously.[40]

**Antifungal (YNG) Assay.** The assay was performed as described previously.[41]

## Electronic Supplementary Material

Supplementary material is available in the online version of this article at http://dx.doi.org/ 10.1007/s13659-012-0020-5 and is accessible for authorized users.

## Acknowledgments

We are grateful to the members of the analysis group of our institute for the measurement of spectroscopic data. This work was supported by the 973 Program of Ministry of Science and Technology of China (2011CB915503), the Fourteenth Candidates of the Young Academic Leaders of Yunnan Province (Min XU, 2011CI044), and NSFC 31060054.

**Open Access** This article is distributed under the terms of the Creative Commons Attribution License which permits any use, distribution, and reproduction in any medium, provided the original author(s) and source are credited.

## Electronic supplementary material

Supplementary material, approximately 702 KB.
