# Supplementary material for: Flavonoid oligomers from Chinese dragon’s blood, the red resins of Dracaena cochinchinensis
Source: Nat Prod Bioprospect. 2012 Apr 11;2(3):111–6. doi: 10.1007/s13659-012-0020-5 (PMC4131598; doi:10.1007/s13659-012-0020-5)
Supplement: Supplementary file 1 — Supplementary material, approximately 702 KB. [file 13659_2012_20_MOESM1_ESM.pdf]

Electronic Supplementary Material

Flavonoid oligomers from Chinese dragon's blood, the red resins of *Dracaena cochinchinensis*

Qing-An ZHENG,[a] Min XU,[a,]* Chong-Ren YANG,[a,b] Dong WANG,[a] Hai-Zhou LI,[a] Hong-Tao ZHU,[a] and Ying-Jun ZHANG[a,]*

[a]State Key Laboratory of Phytochemistry and Plant Resources in West China, Kunming Institute of Botany, Chinese Academy of Sciences, Kunming 650201, China

[b]Weihe Biotech Laboratory, Yuxi 653100, China

Received 5 March 2012; Accepted 27 March 2012

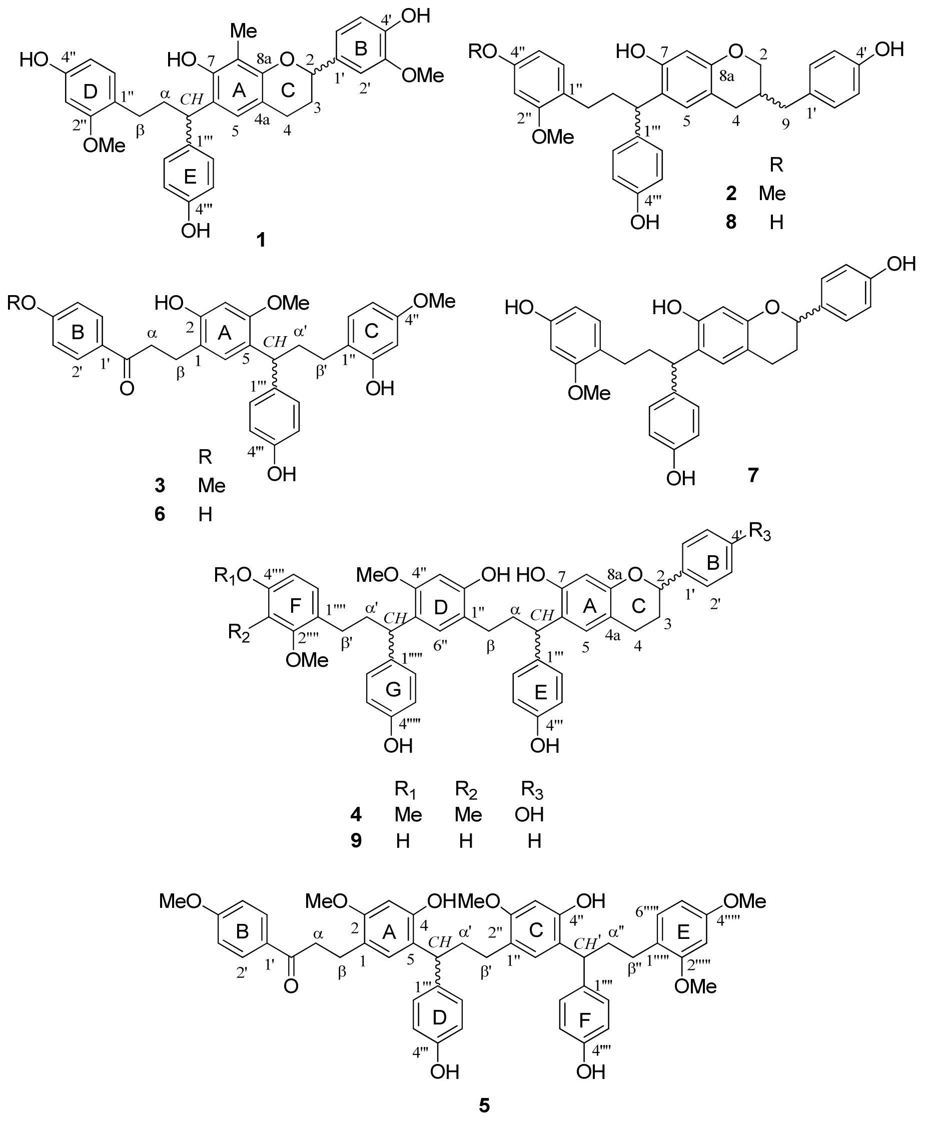

*To whom correspondence should be addressed. E-mail:zhangyj@mail.kib.ac.cn (Y.J. Zhang); xumin@mail.kib.ac.cn (X. Min).

S1 Supplementary data contents page

Title: **Flavonoid Oligomers from Chinese Dragon's Blood, the Red Resins of *Dracaena cochinchinensis***

Authors: Qing-An Zheng, Min Xu, Chong-Ren Yang, Dong Wang, Hai-Zhou Li, Hong-Tao Zhu, and Ying-Jun Zhang

Address: Kunming Institute of Botany, Chinese Academy of Sciences, Kunming 650204, China; Weihe Biotech Laboratory, 653100, Yuxi, Yunnan, P. R. China

S2 ^{1}H NMR spectrum of cochinchinenin D (**1**) in CD_3OD

S3 ^{1}H NMR spectrum of cochinchinenin D (**1**) in CD_3OD

S4 ^{13}C NMR spectrum for cochinchinenin D (**1**) in CD_3OD

S5 HMQC spectrum of cochinchinenin D (**1**) in CD_3OD

S6 ^{1}H-^{1}H COSY spectrum of cochinchinenin D (**1**) in CD_3OD

S7 HMBC spectrum for cochinchinenin D (**1**) in CD_3OD

S8 ^{1}H NMR spectrum of cochinchinenin E (**2**) in CD_3OD

S9 ^{13}C NMR spectrum of cochinchinenin E (**2**) in CD_3OD

S10 HMQC spectrum of cochinchinenin E (**2**) in CD_3OD

S11 ^{1}H-^{1}H COSY spectrum of cochinchinenin E (**2**) in CD_3OD

S12 HMBC spectrum of cochinchinenin E (**2**) in CD_3OD

S13 ^{1}H NMR spectrum for cochinchinenin F (**3**) in CD_3OD

S14 ^{13}C NMR spectrum for cochinchinenin F (**3**) in CD_3OD

S15 ^{1}H NMR spectrum for cochinchinenin G (**4**) in CD_3OD

S16 ^{13}C NMR spectrum for cochinchinenin G (**4**) in CD_3OD

S17 ^{1}H NMR spectrum of cochinchinenin H (**5**) in CD_3OD

S18 ^{13}C NMR spectrum of cochinchinenin H (**5**) in CD_3OD

S19 HMQC spectrum of cochinchinenin H (**5**) in CD_3OD

S20 ^{1}H-^{1}H COSY spectrum of cochinchinenin H (**5**) in CD_3OD

S21 HMBC spectrum of cochinchinenin H (**5**) in CD_3OD

S22 ROESY spectrum of cochinchinenin H (**5**) in CD_3OD

S23 ^{1}H-^{1}H COSY and selected HMBC correlations of compounds **2** and **3**

S24 ^{1}H-^{1}H COSY and selected HMBC correlations of compounds **4** and **5**

S2 ^{1}H NMR spectrum of cochinchinenin D (1) in CD_3OD

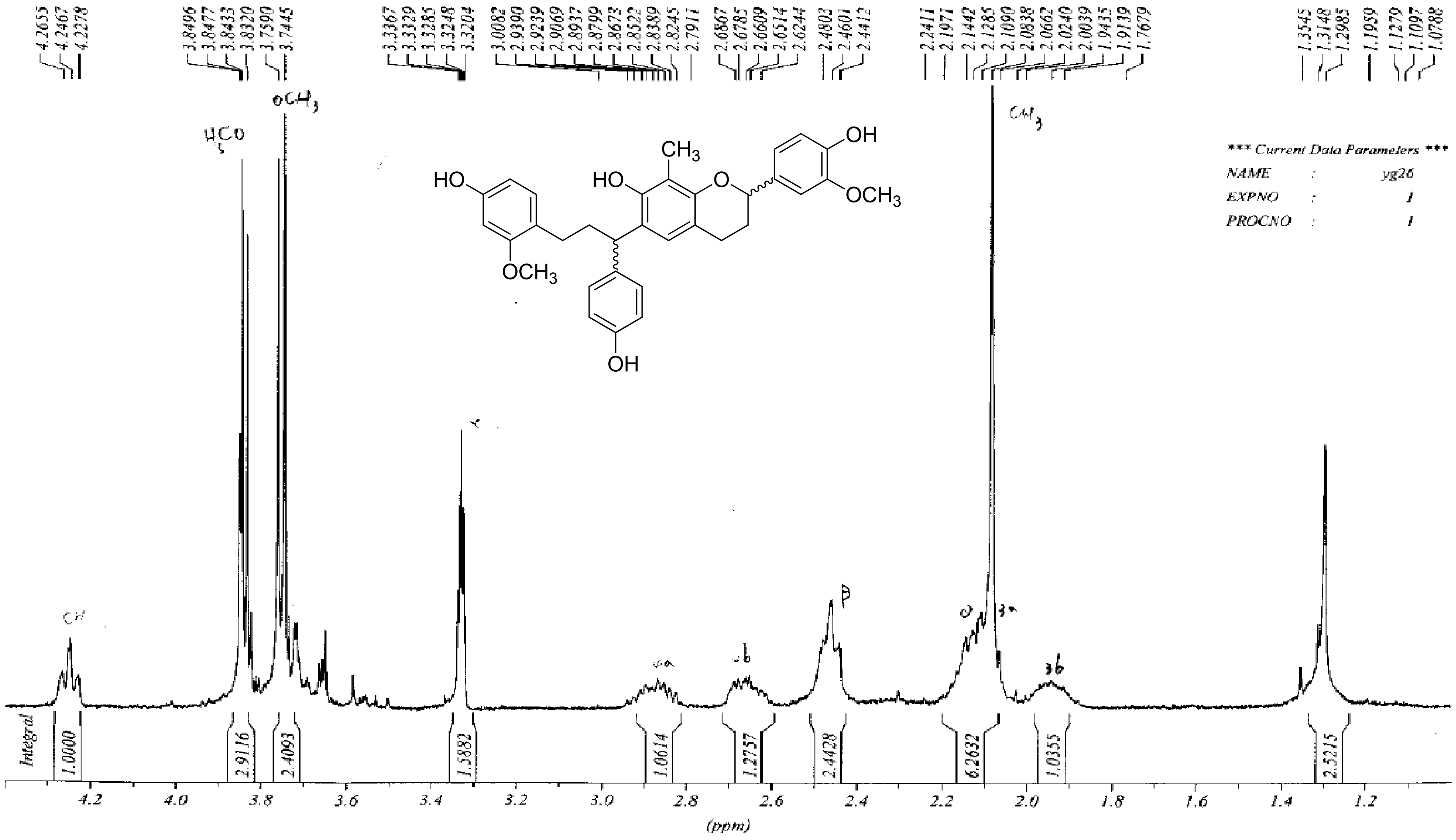

S3 ^{1}H NMR spectrum of cochinchinenin D (1) in CD_3OD

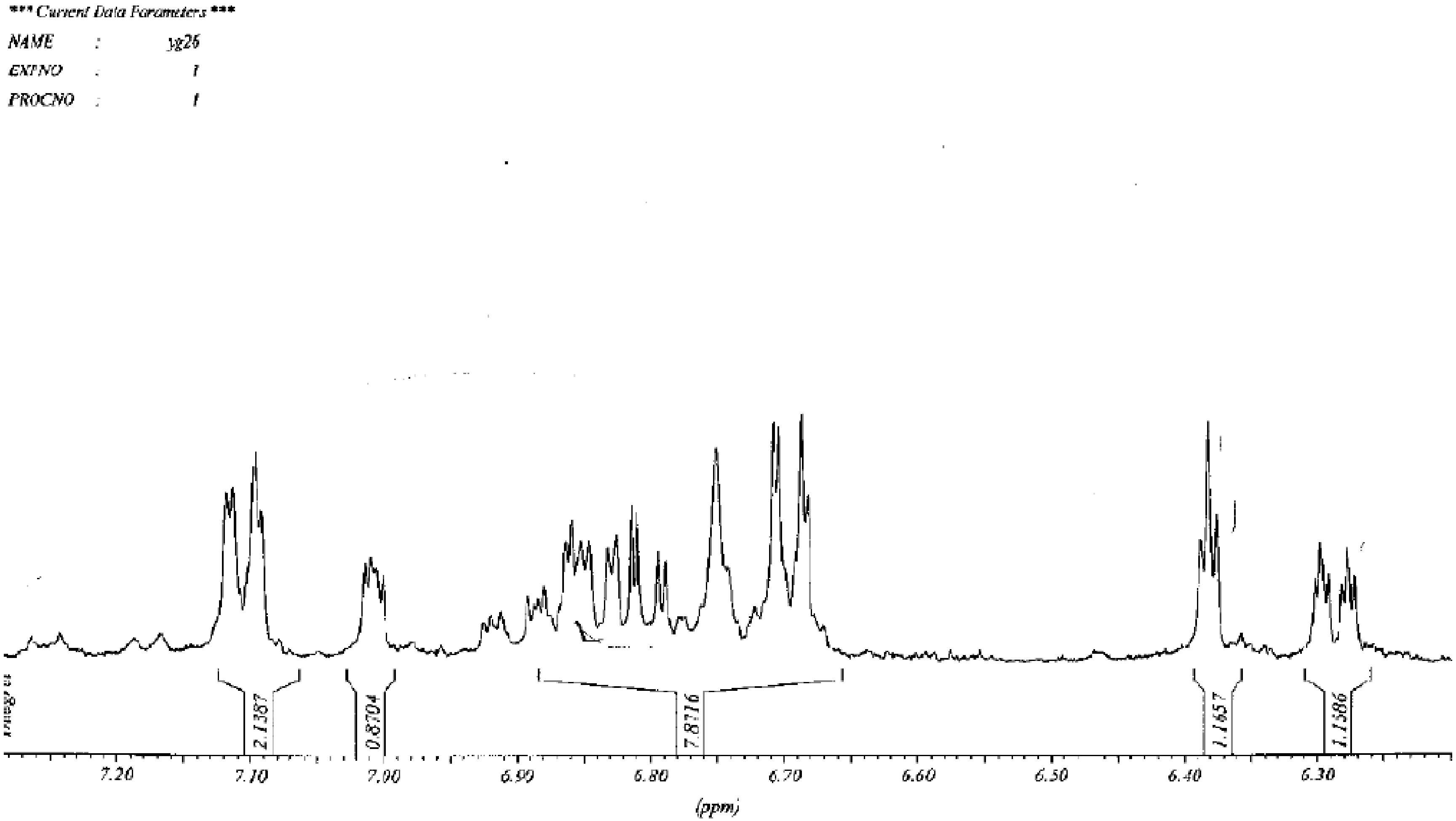

S4 ^{13}C NMR spectrum for cochinchinenin D (1) in CD_3OD

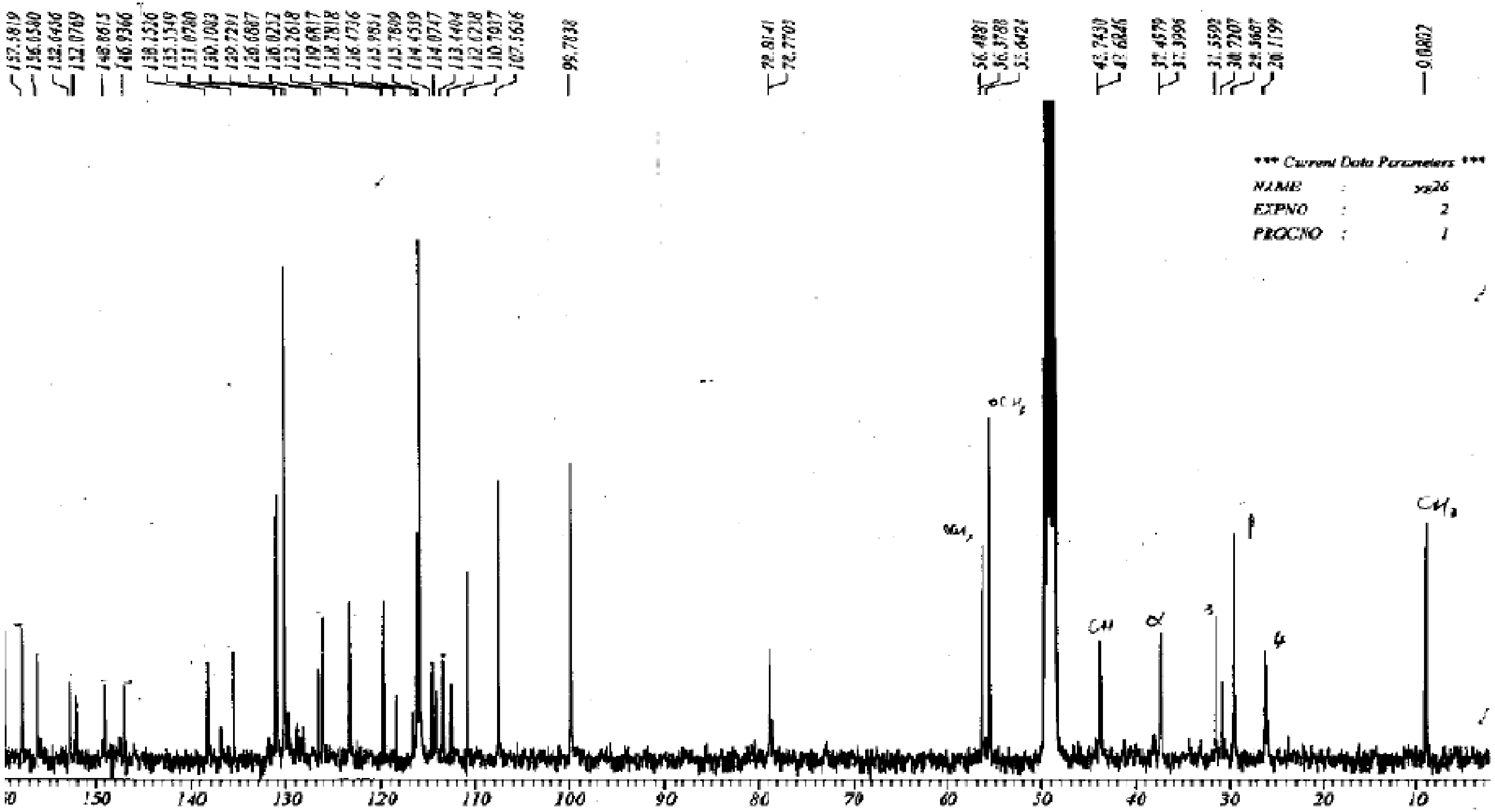

S5 HMQC spectrum of cochinchinenin D (1) in CD_3OD

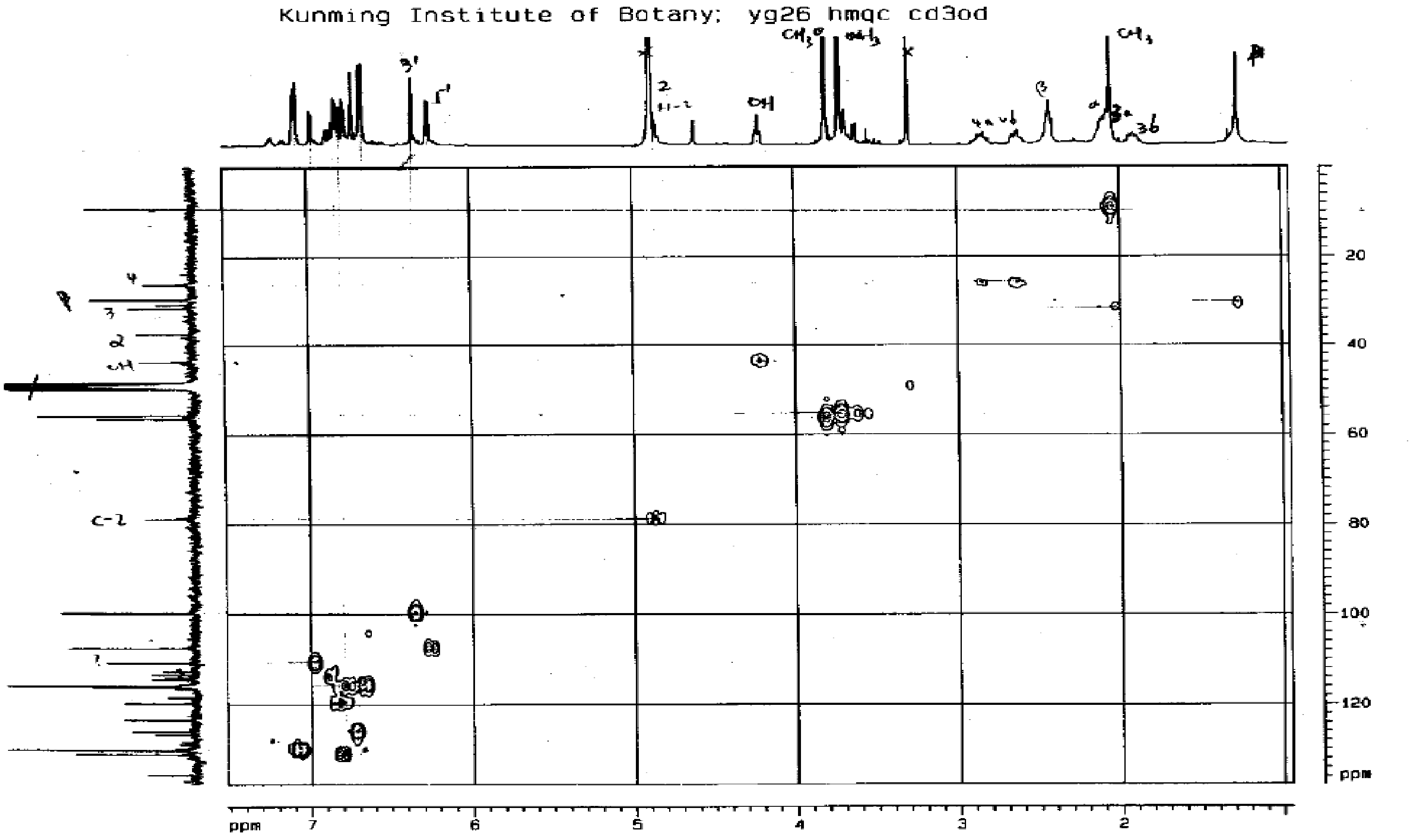

S6 ^{1}H-^{1}H COSY spectrum for cochinchinenin D (1) in CD_3OD

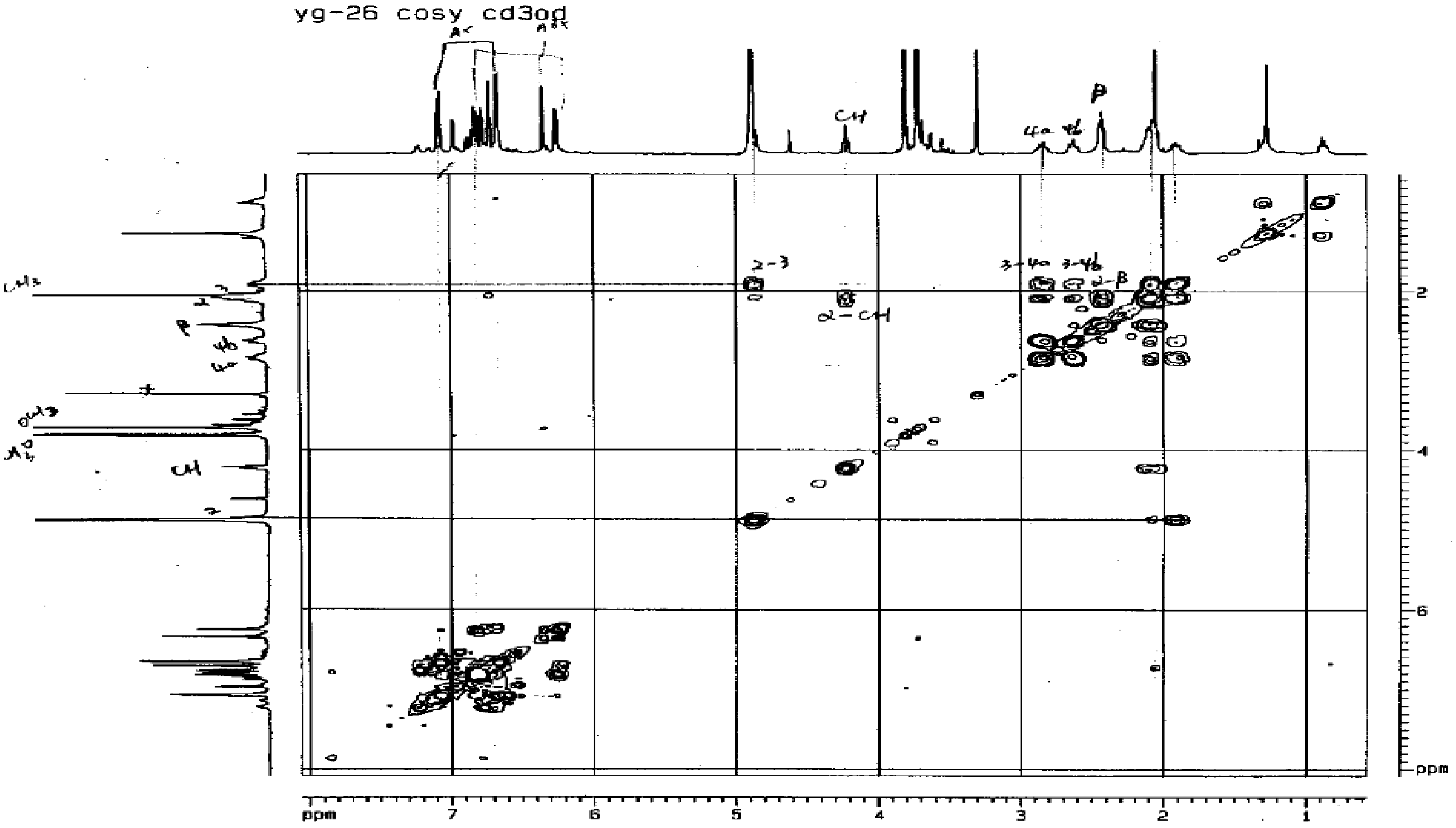

S7 HMBC spectrum for cochinchinenin D (1) in CD_3OD

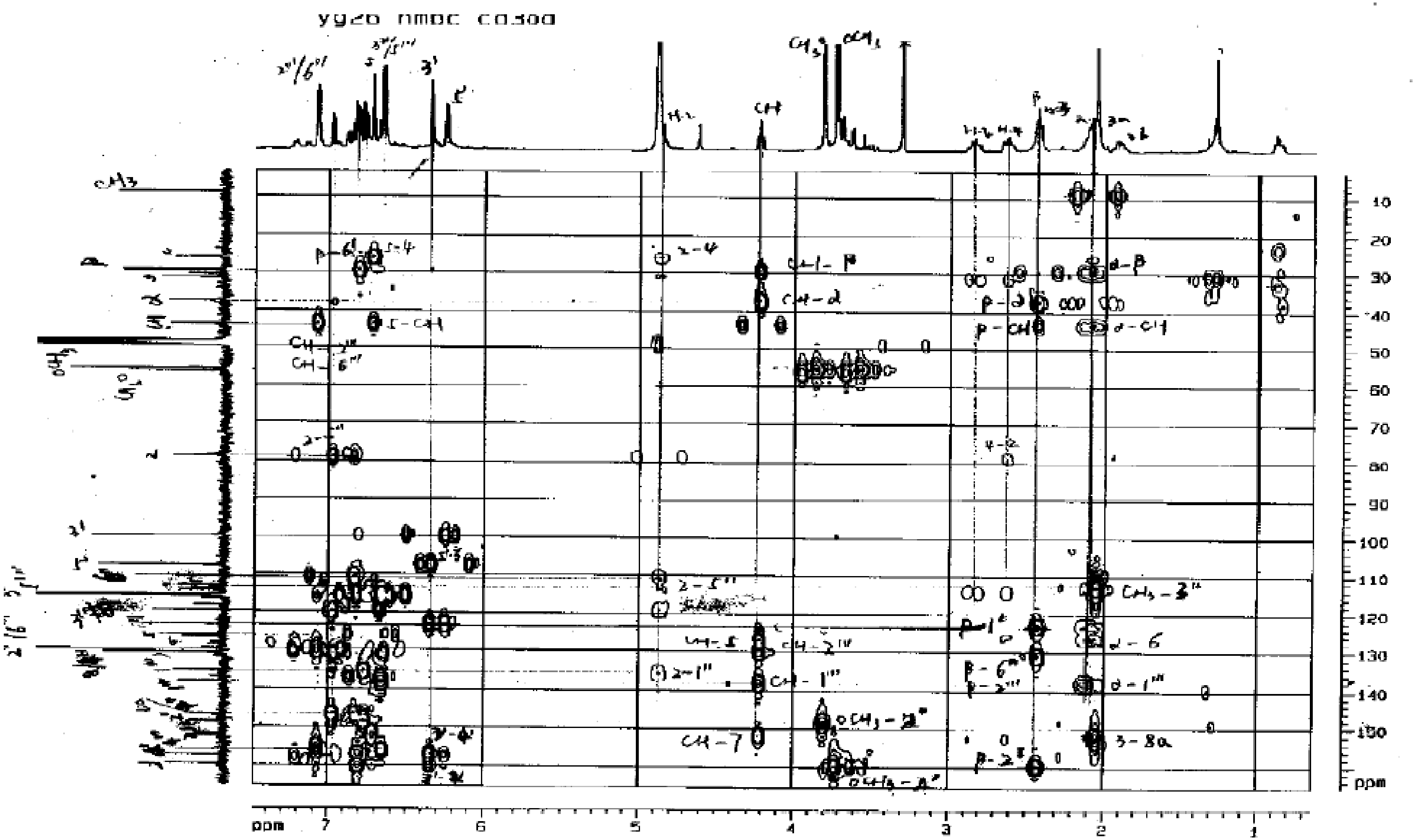

S8 ^{1}H NMR spectrum of cochinchinenin E (2) in CD_3OD

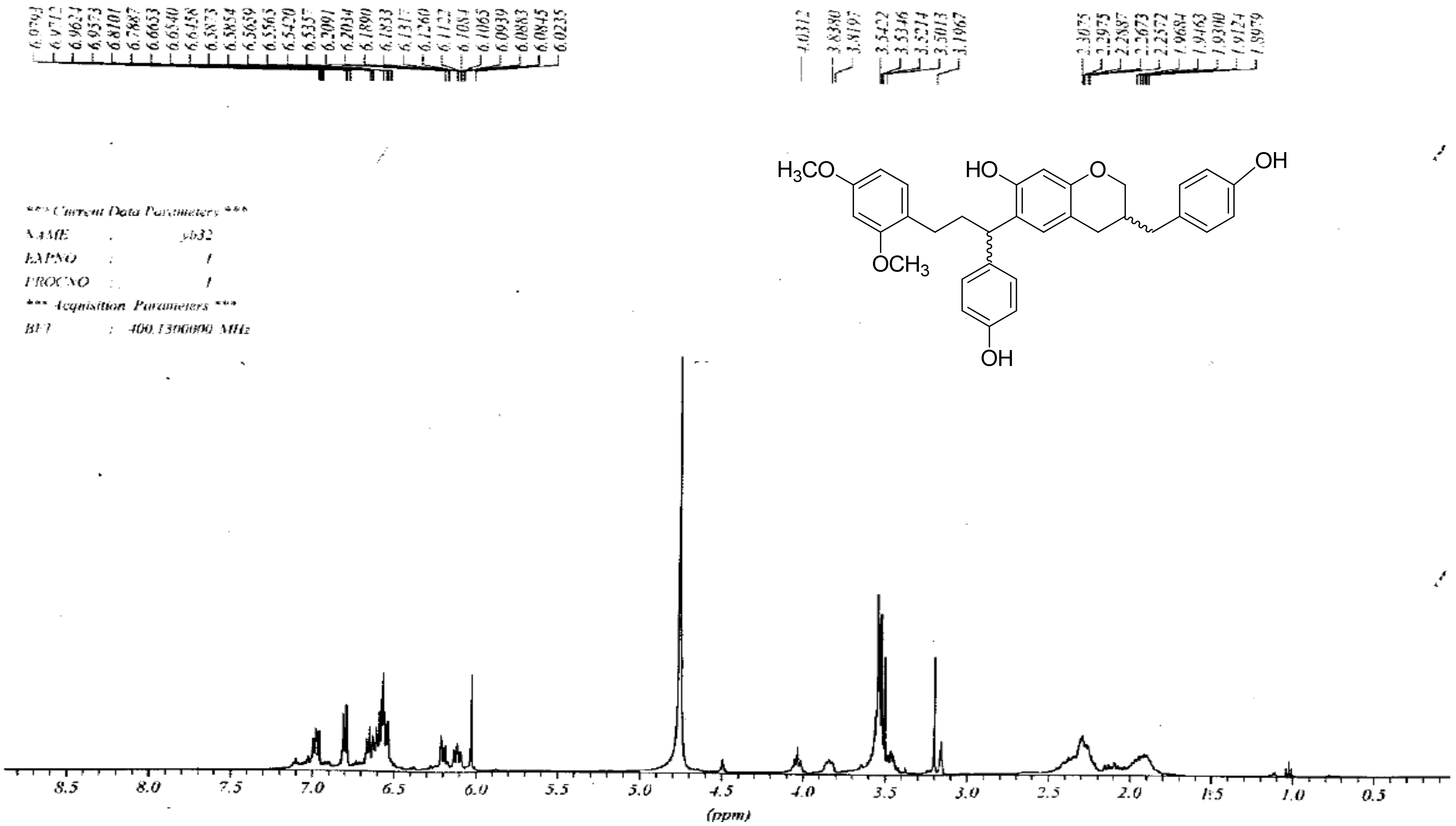

S9 ^{13}C NMR spectrum of cochinchinenin E (2) in CD_3OD

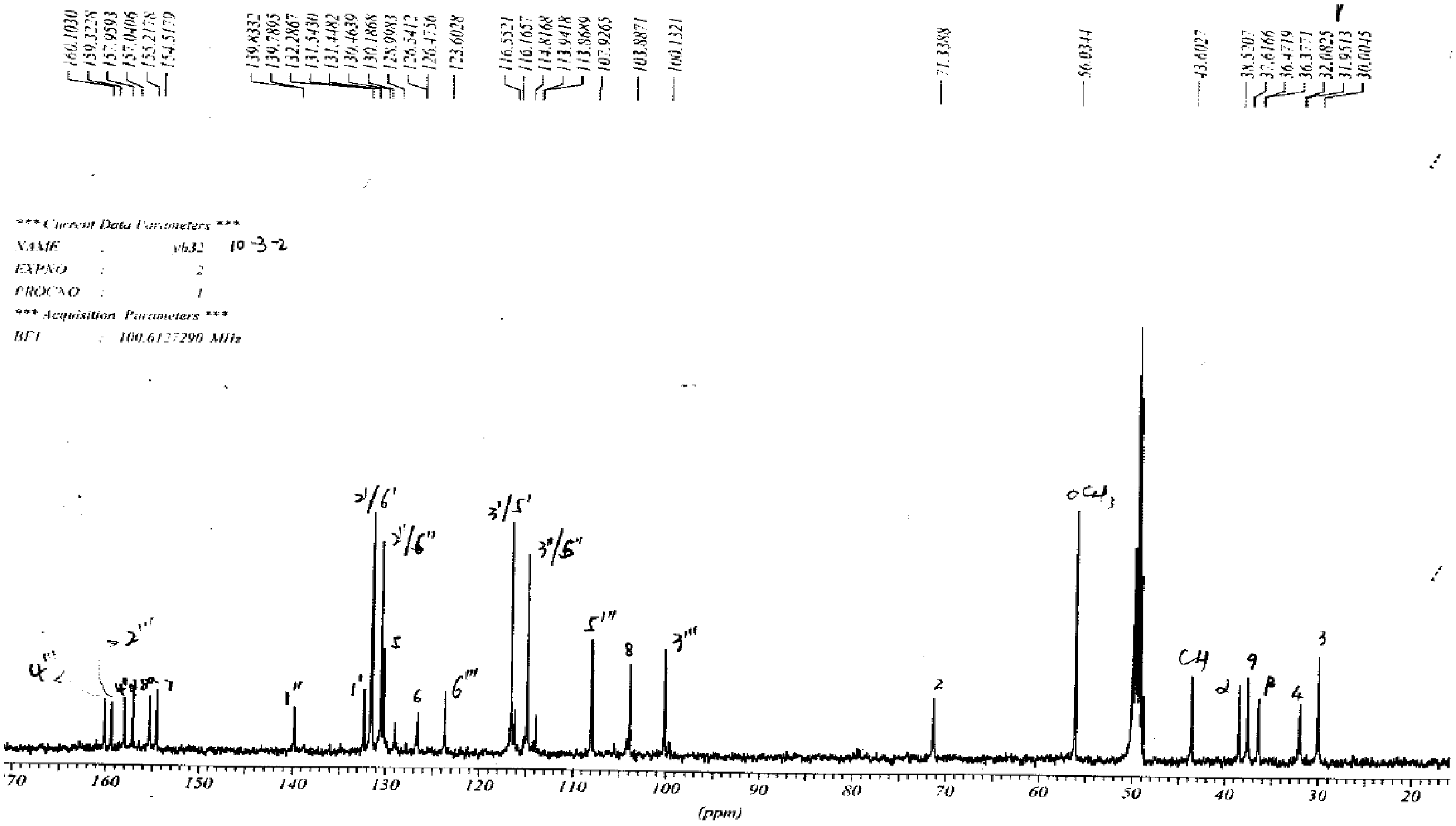

S10 HMQC spectrum of cochinchinenin E (2) in CD_3OD

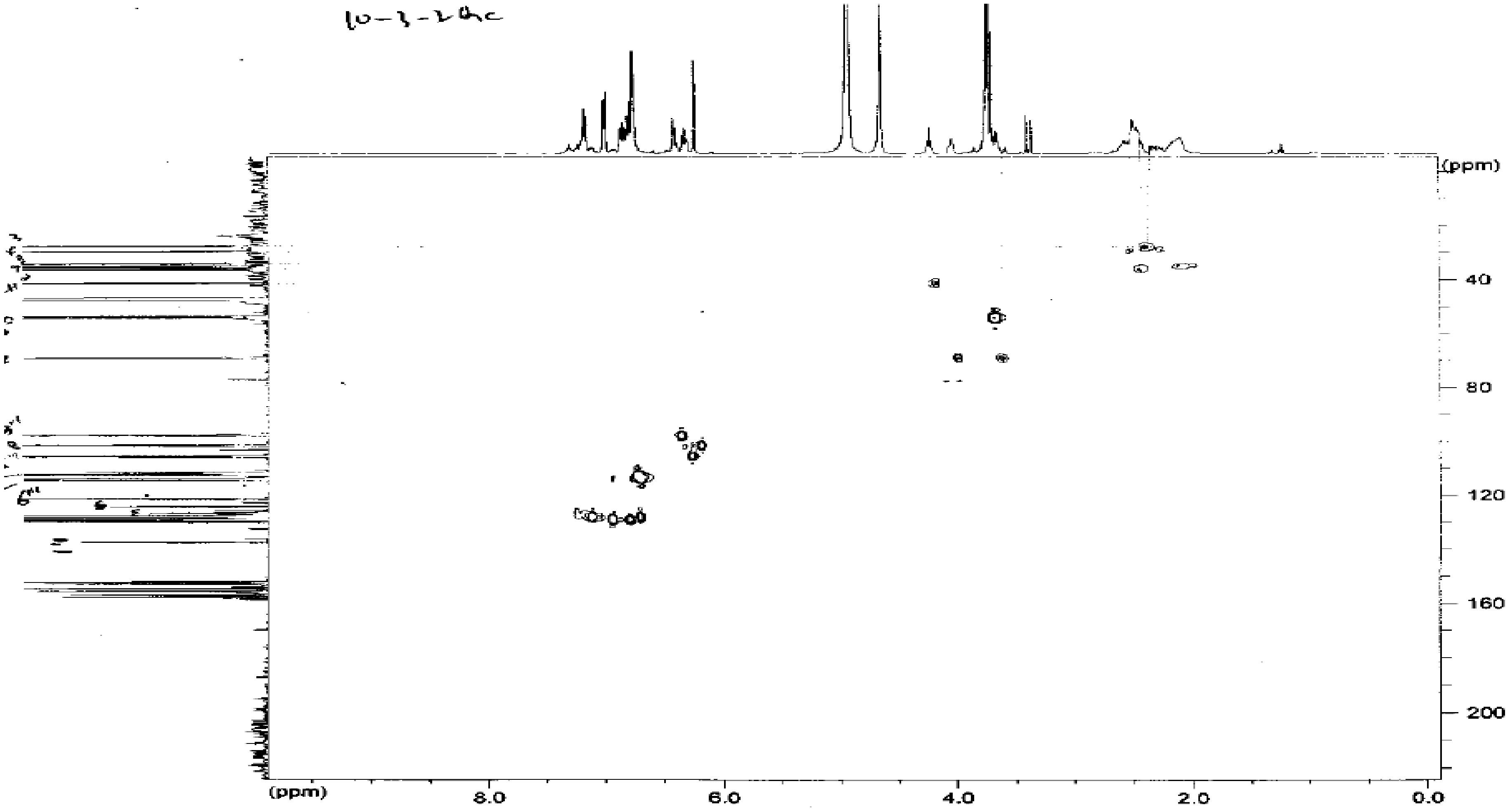

S11 ^{1}H-^{1}H COSY spectrum of cochinchinenin E (2) in CD_3OD

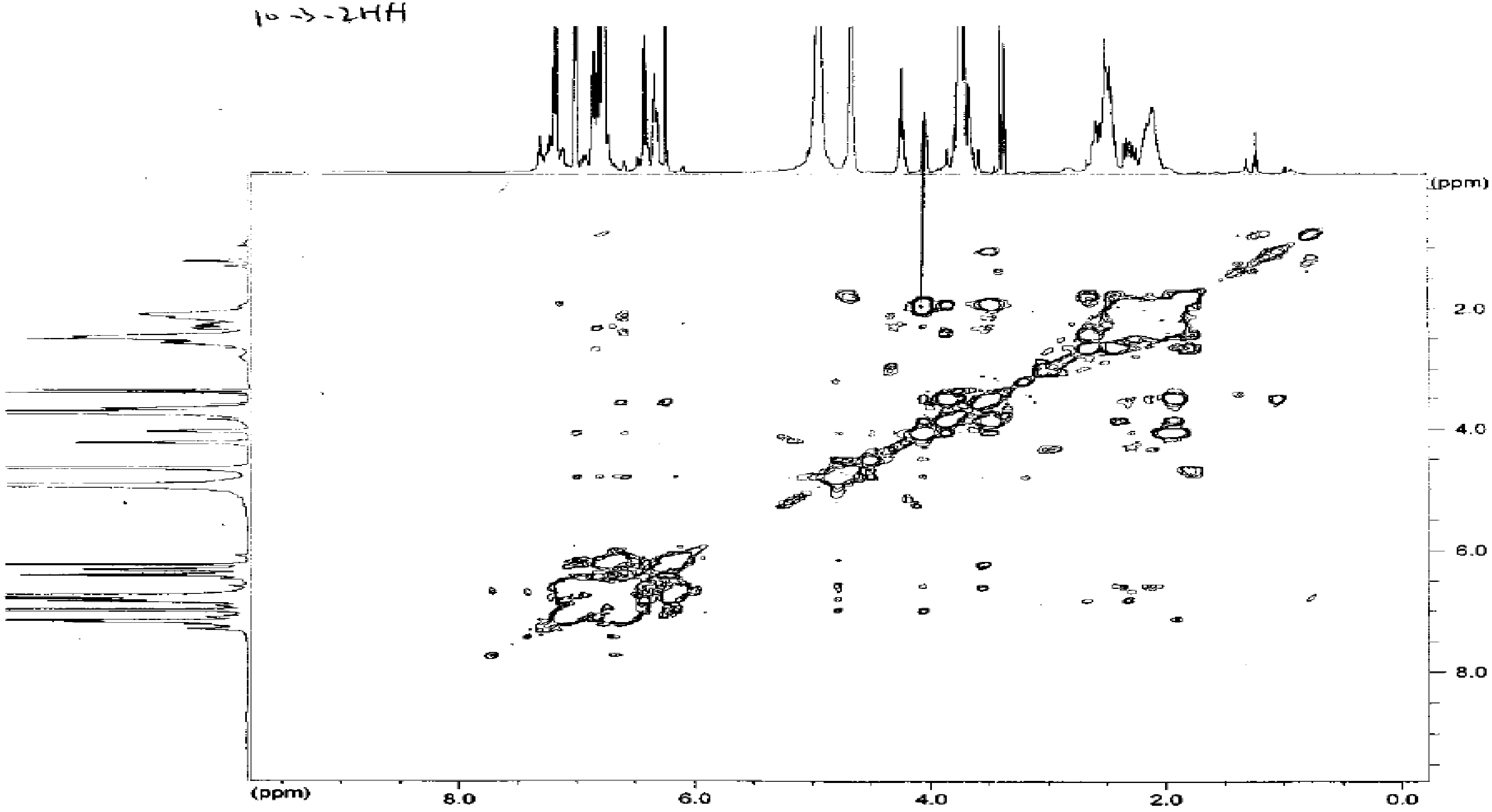

S12 HMBC spectrum of cochinchinenin E (2) in CD_3OD

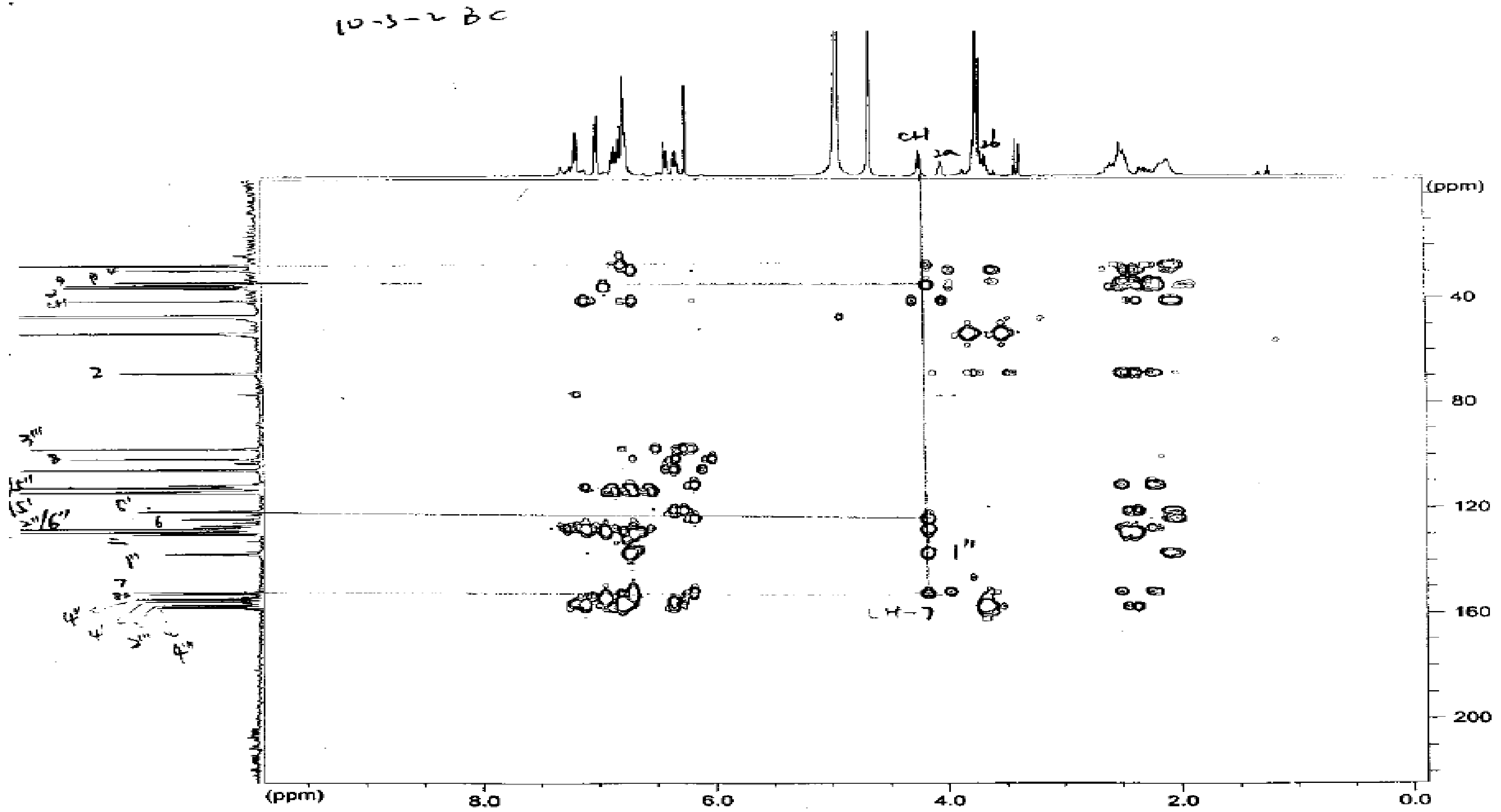

S13 ^{1}H NMR spectrum for cochinchinenin F (3) in CD_3OD

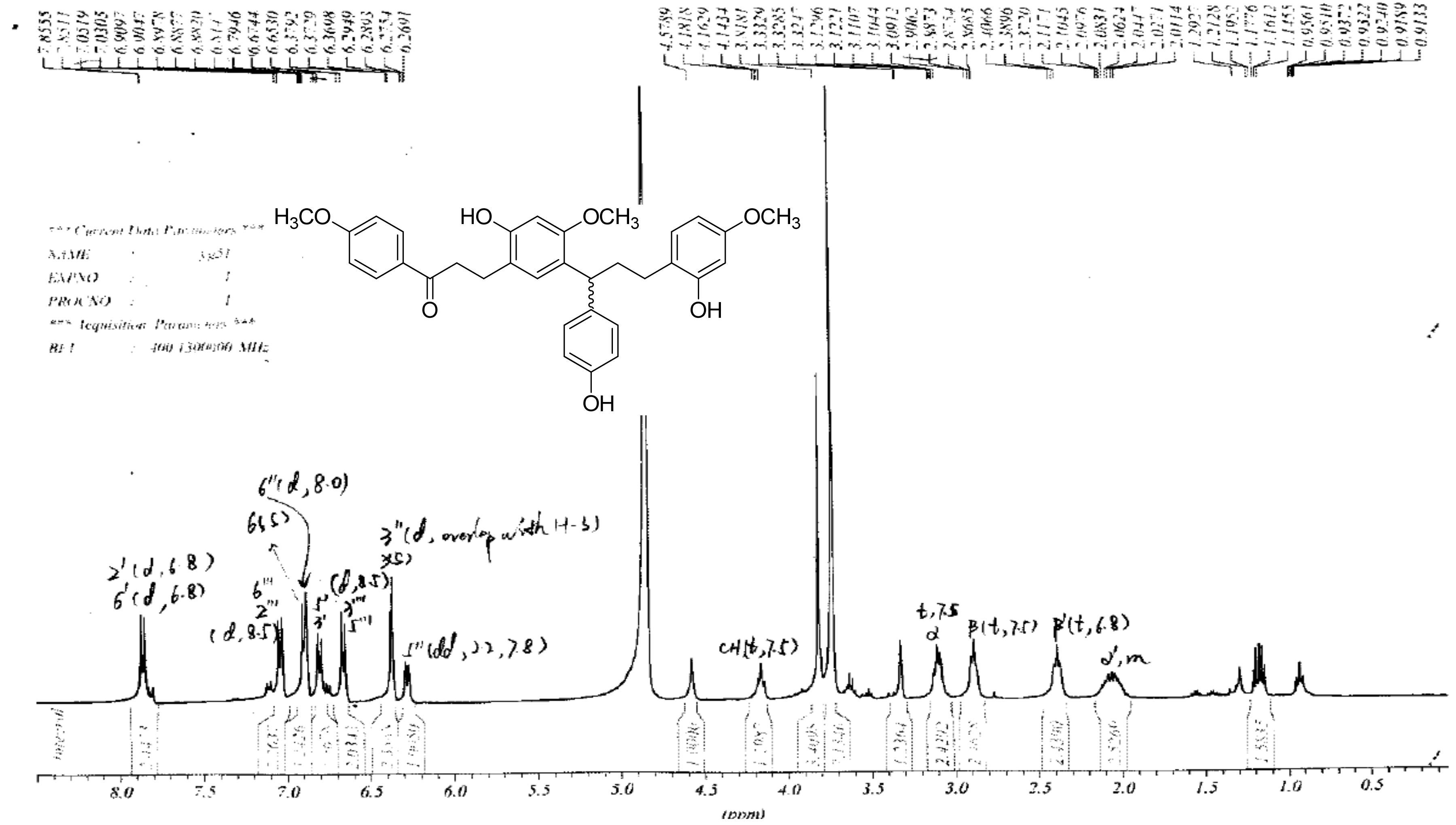

S14 ^{13}C NMR spectrum for cochinchinenin F (3) in CD_3OD

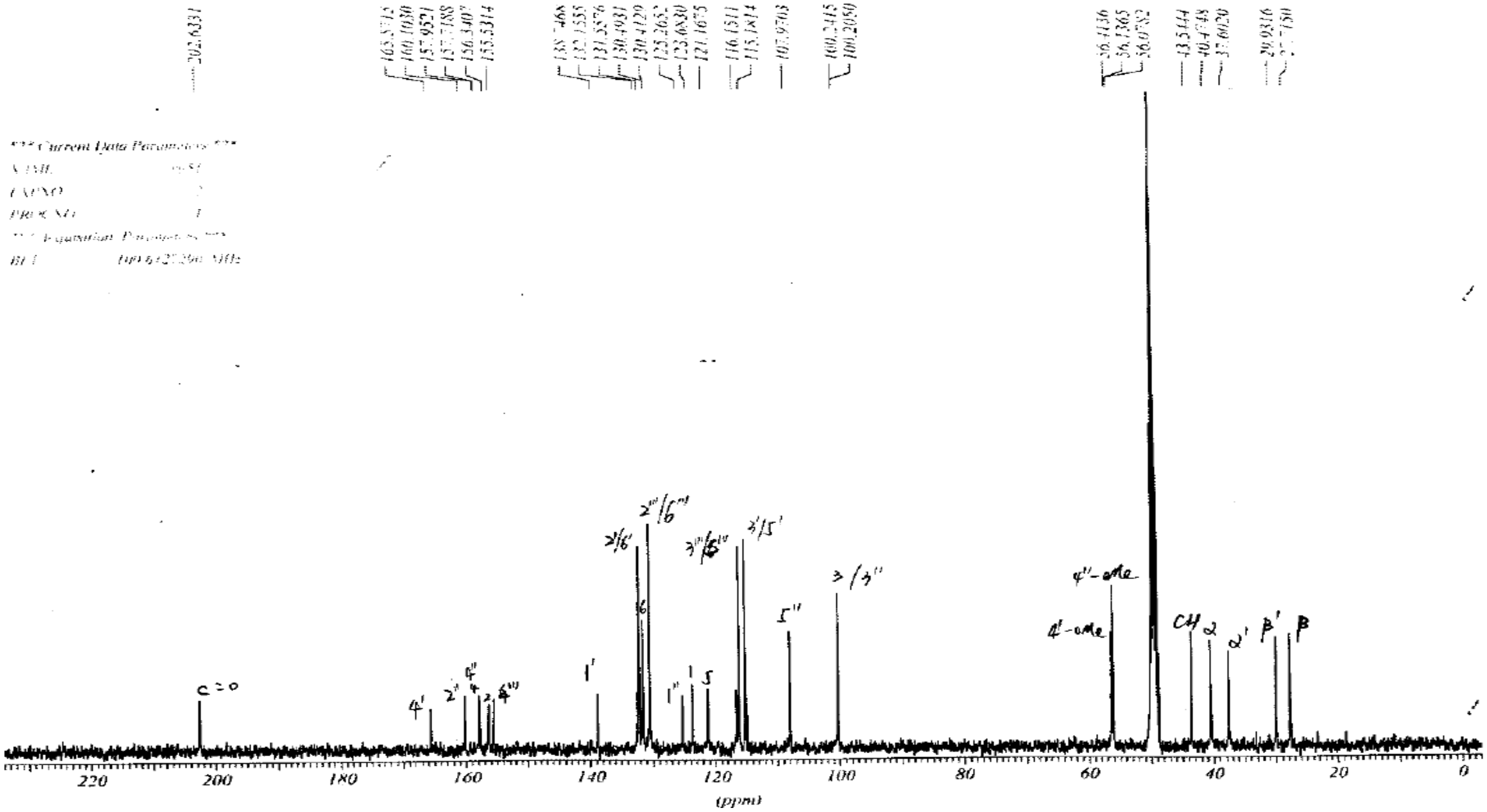

S15 ^{1}H NMR spectrum for cochinchinenin G (4) in CD_3OD

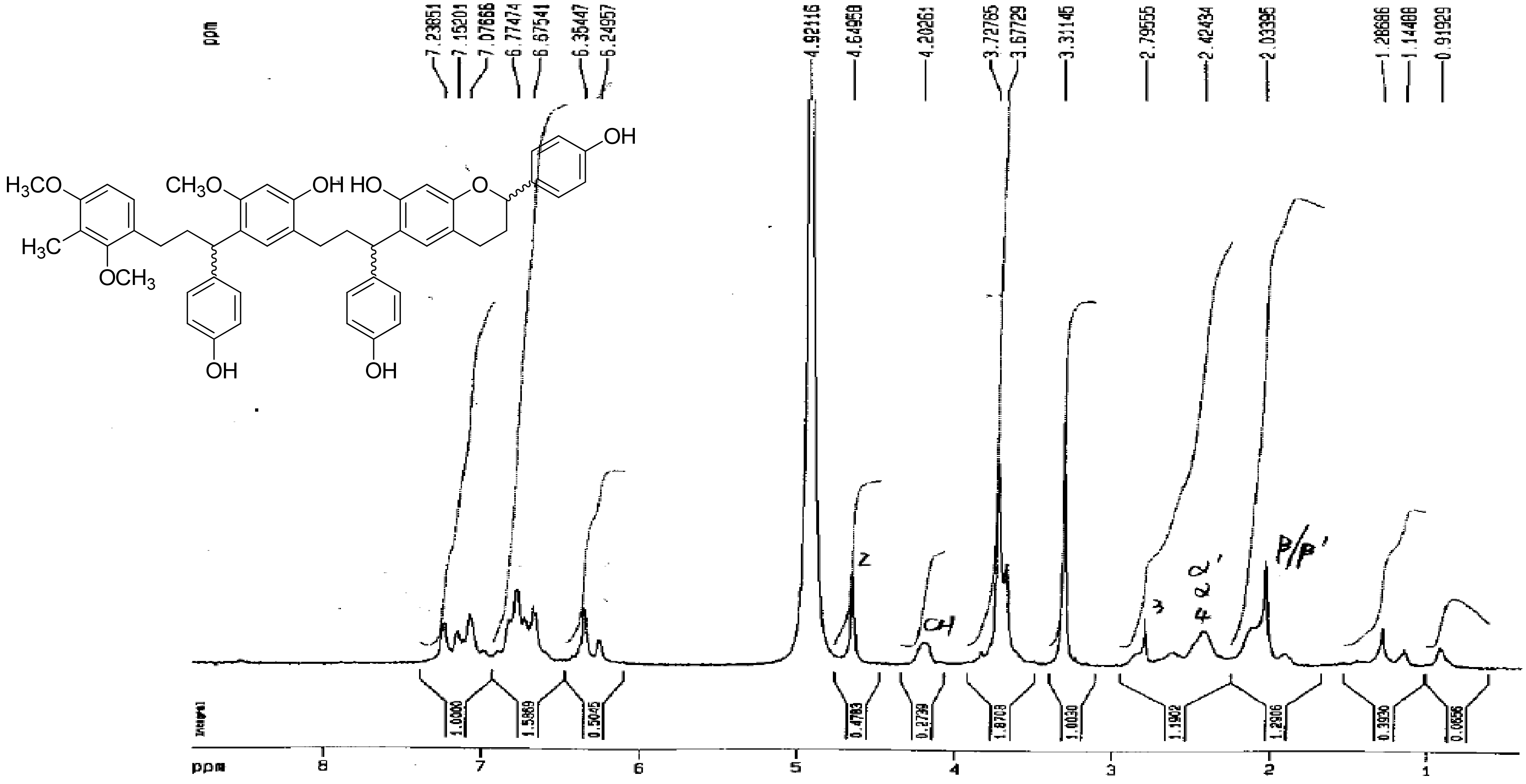

S16 ^{13}C NMR spectrum for cochinchinenin G (4) in CD_3OD

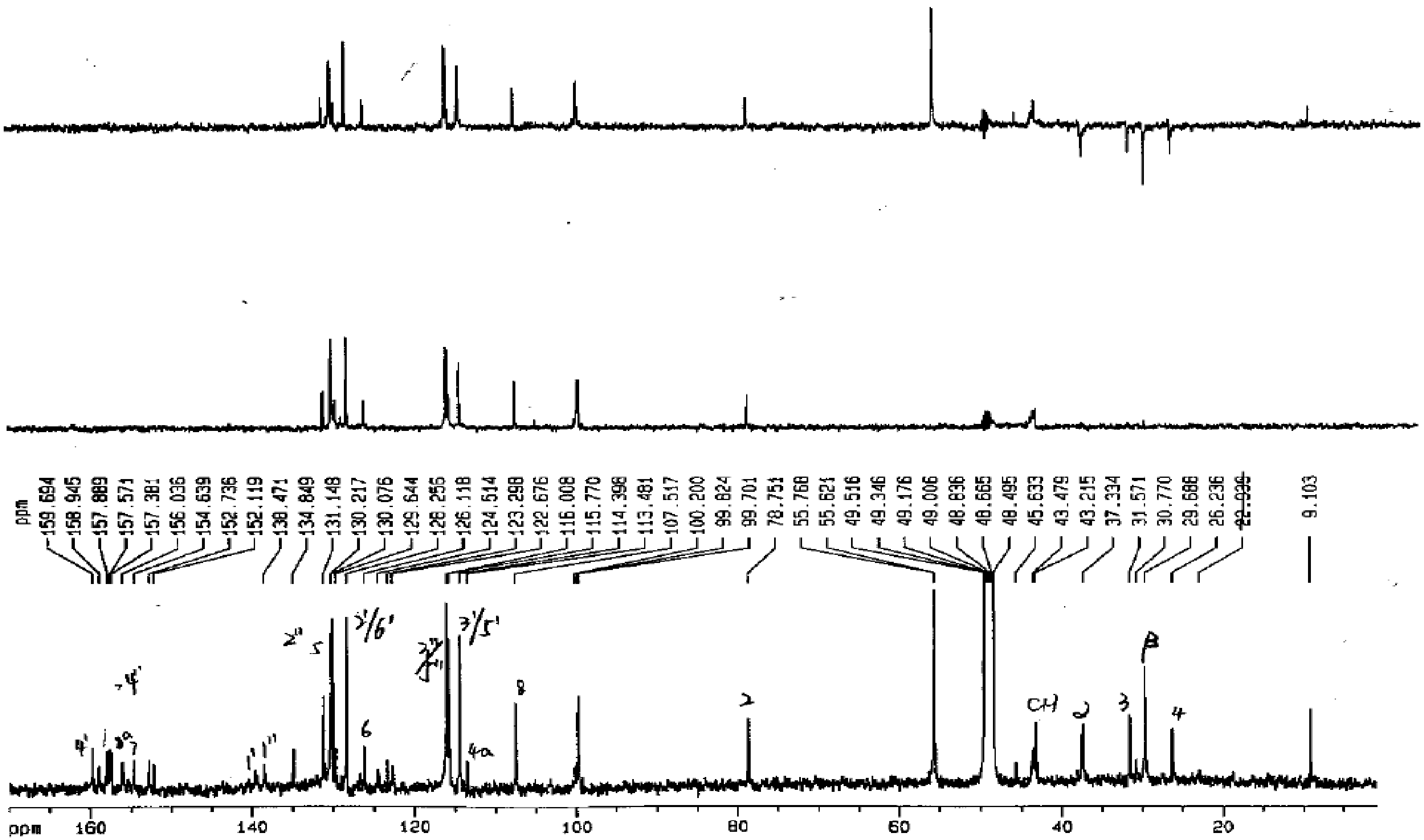

S17 ^{1}H NMR spectrum of cochinchinenin H (5) in CD_3OD

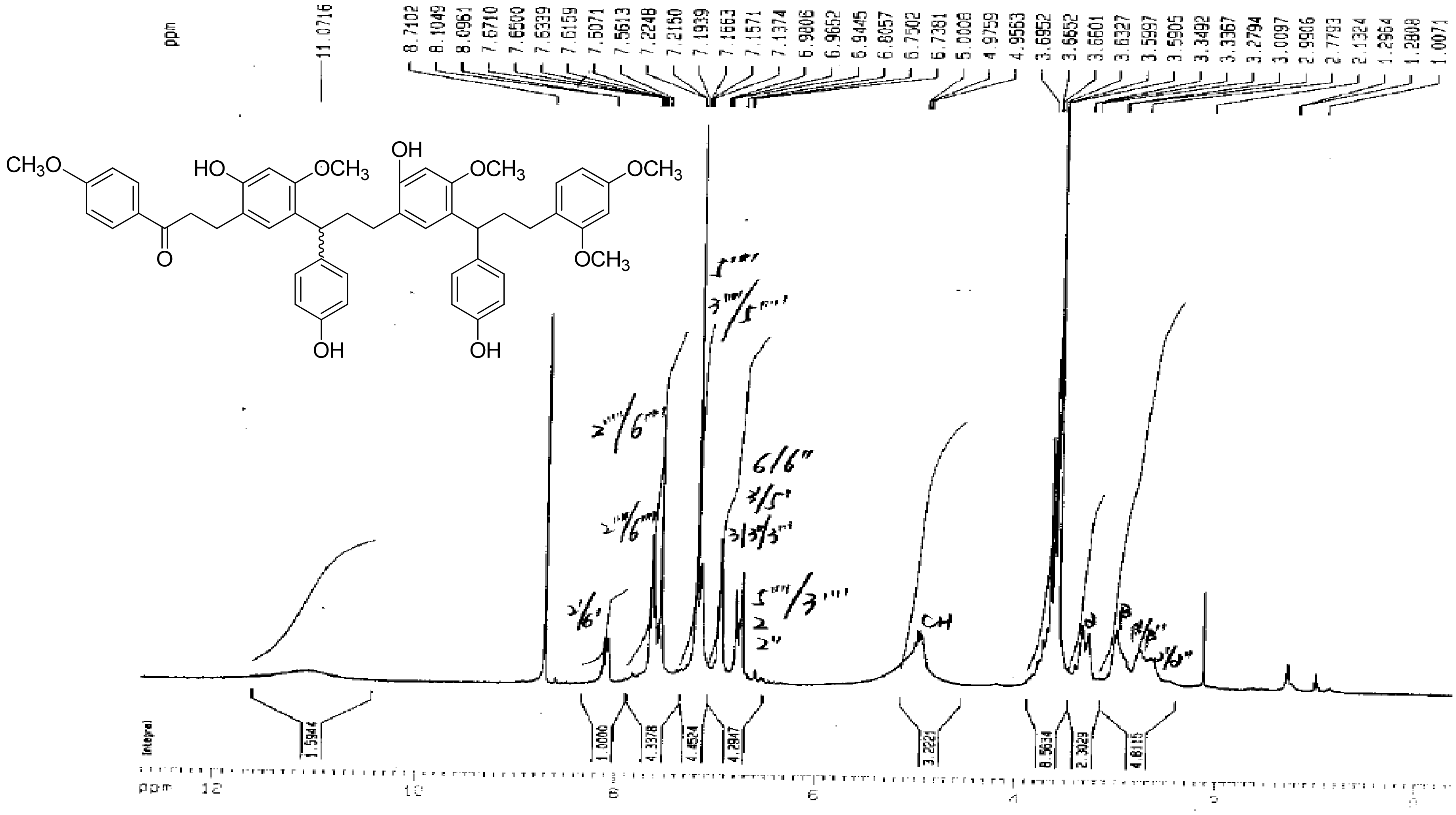

S18 ^{13}C NMR spectrum of cochinchinenin H (5) in CD_3OD

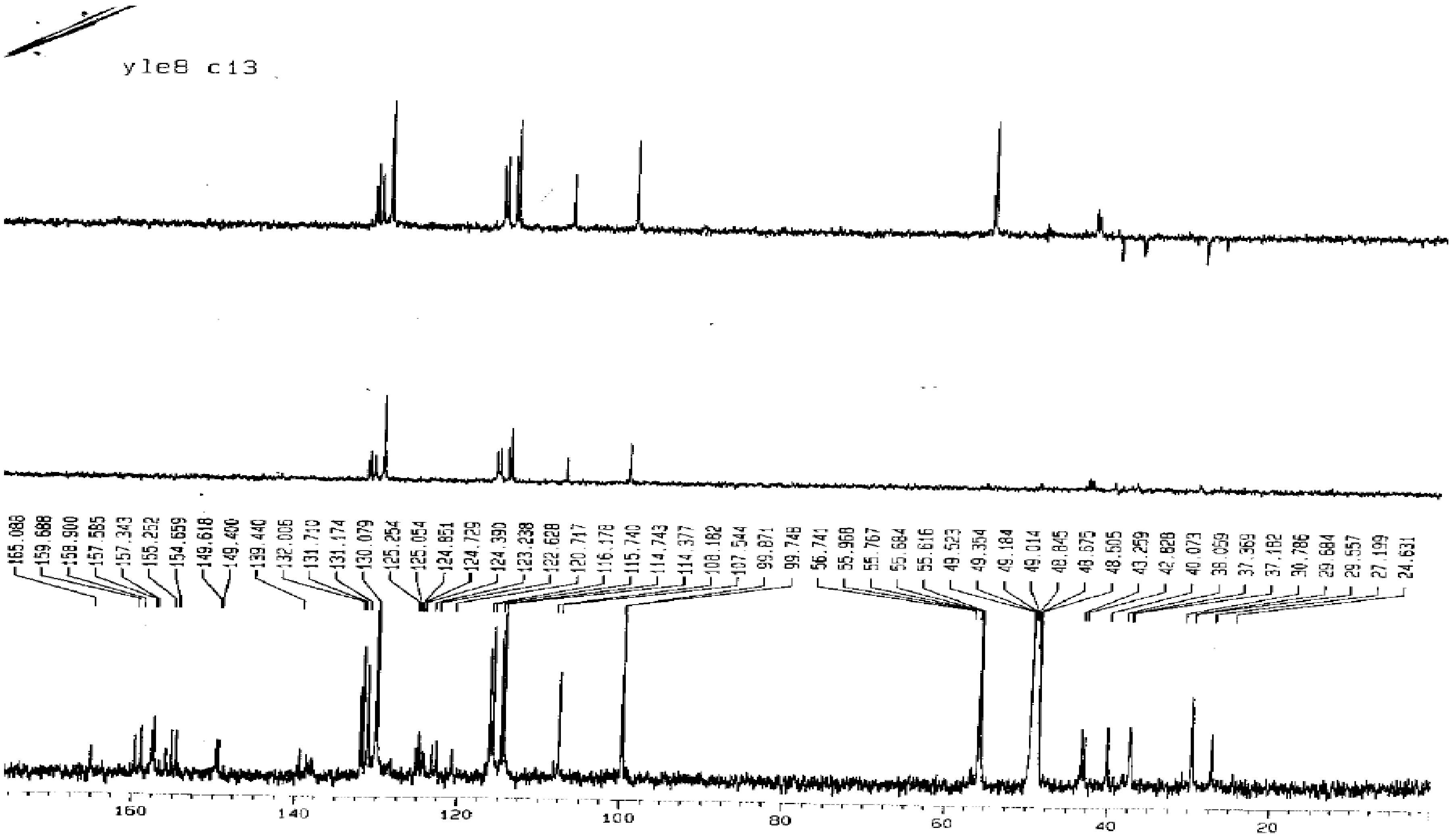

S19 HMQC spectrum of cochinchinenin H (5) in CD_3OD

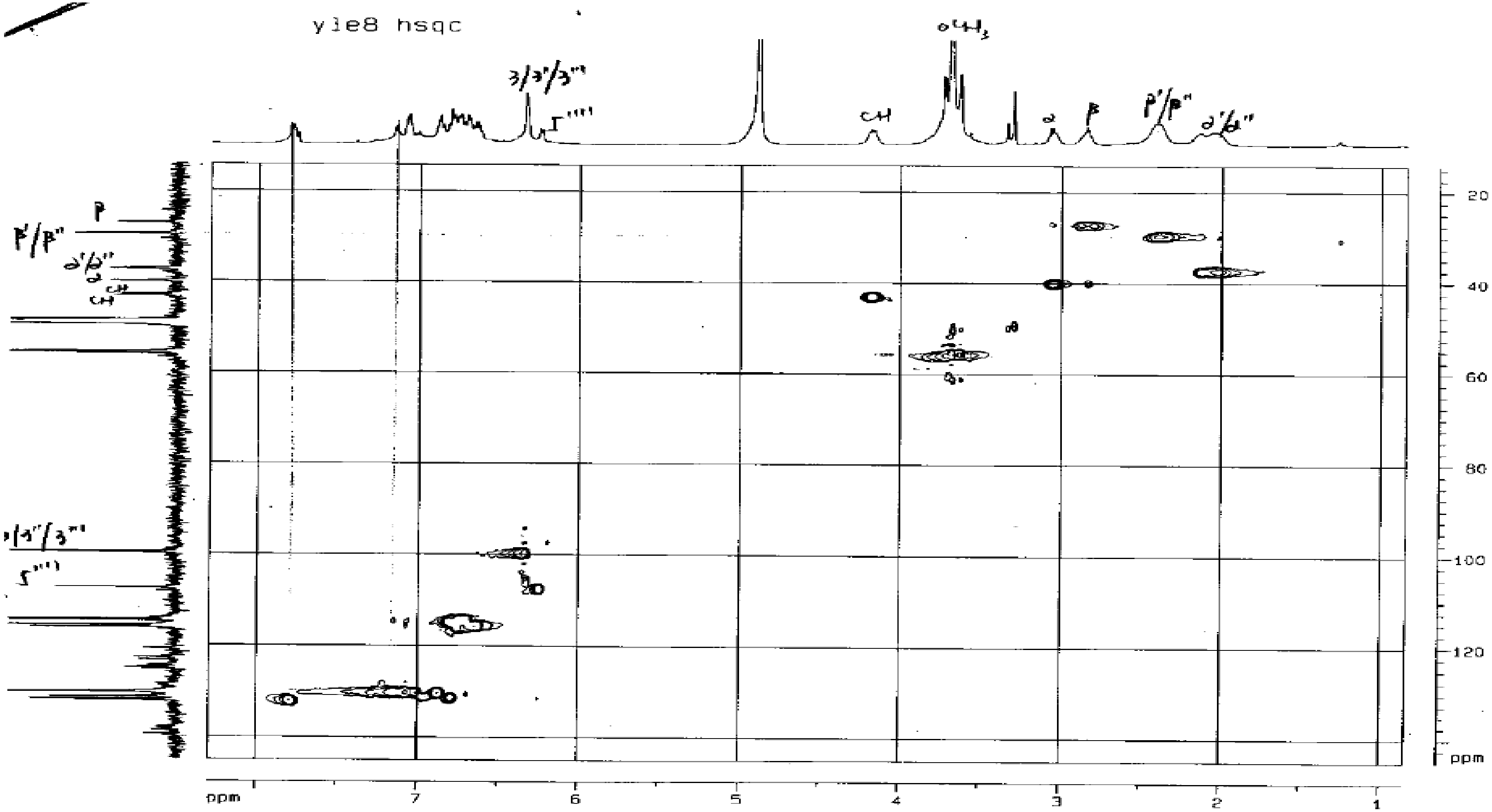

S20 ^{1}H-^{1}H COSY spectrum of cochinchinenin H (5) in CD_3OD

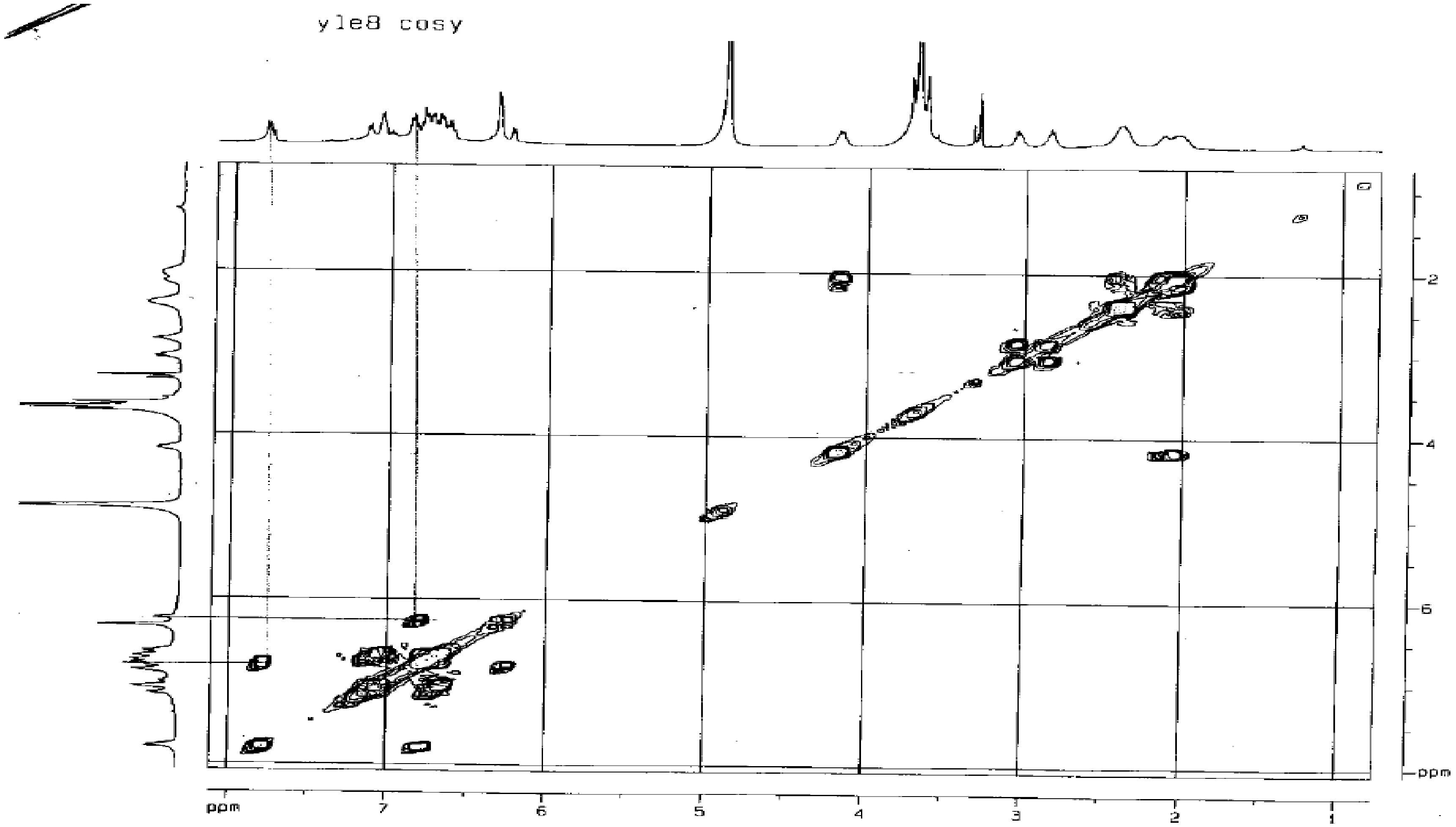

S21 HMBC spectrum of cochinchinenin H (5) in CD_3OD

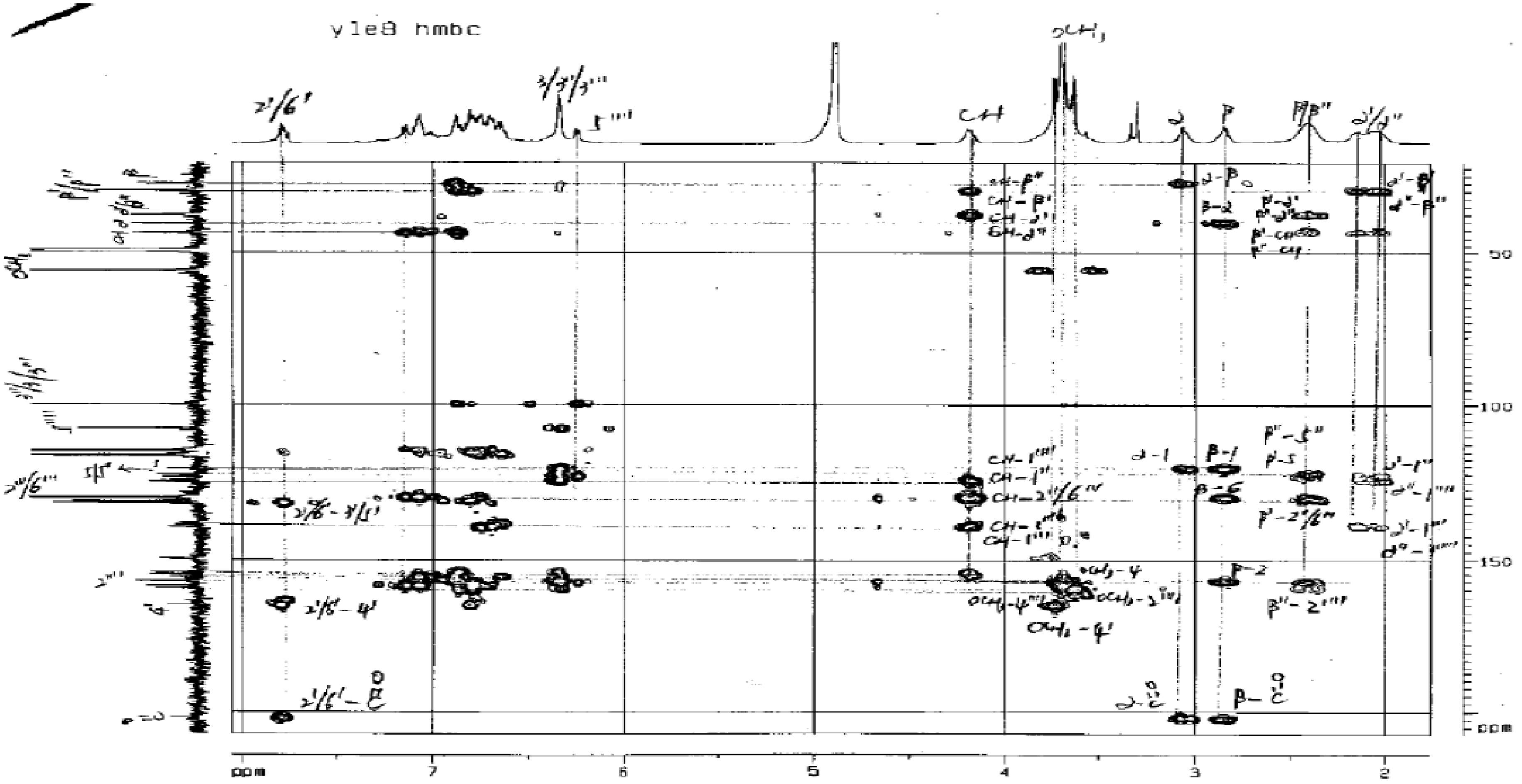

S22 ROESY spectrum of cochinchinenin H (5) in CD_3OD

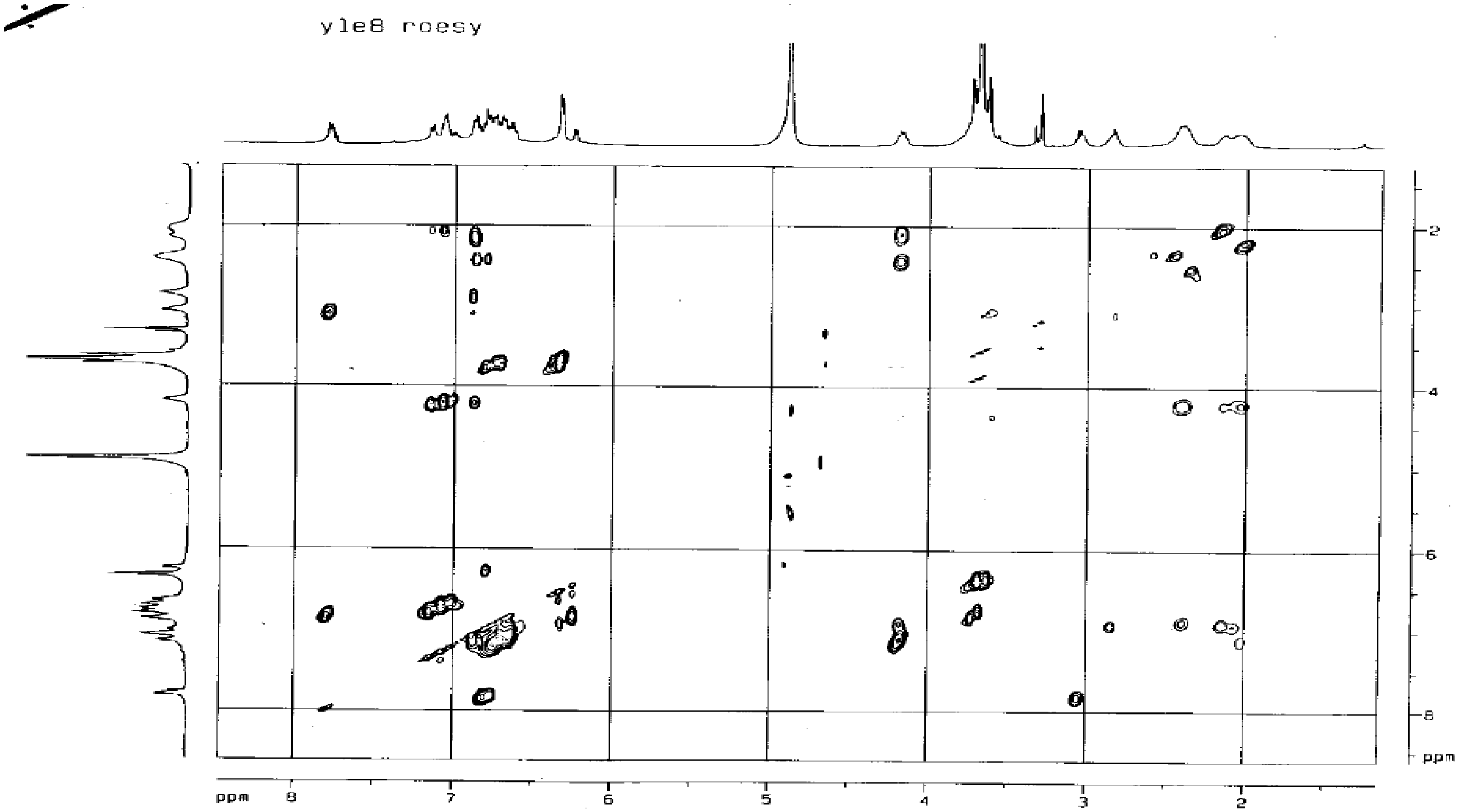

S23 ^{1}H-^{1}H COSY and selected HMBC correlations of compounds 2 and 3

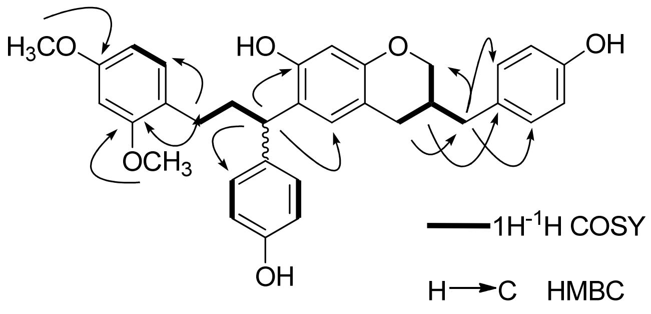

Figure 1. ^{1}H-^{1}H COSY and selected HMBC correlations of **2**

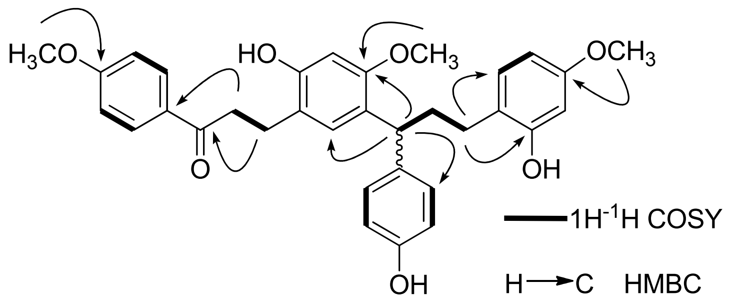

Figure 2. ^{1}H-^{1}H COSY and selected HMBC correlations of **3**

S24 ^{1}H-^{1}H COSY and selected HMBC correlations of compounds 4 and 5

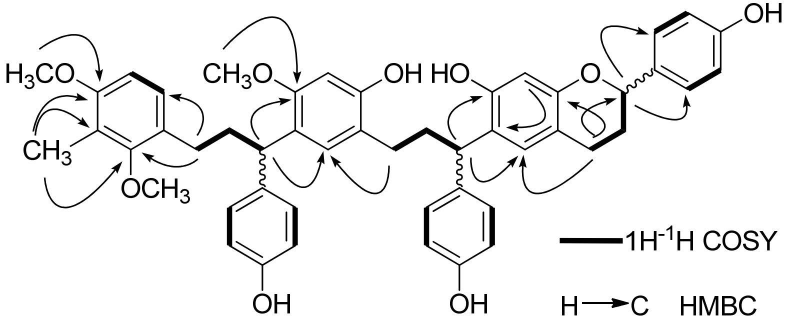

Figure 3. ^{1}H-^{1}H COSY and selected HMBC correlations of **4**

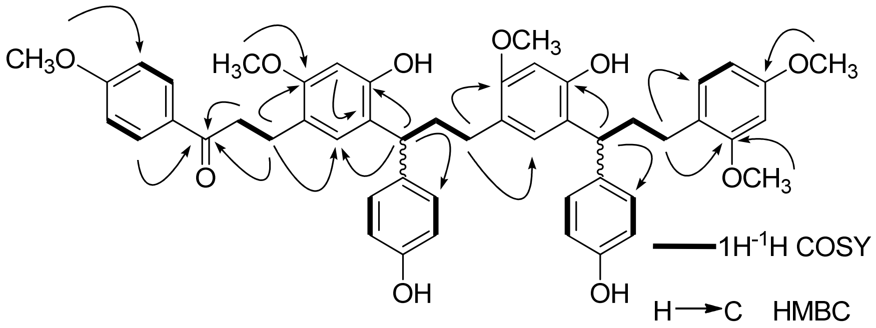

Figure 4. ^{1}H-^{1}H COSY and selected HMBC correlations of **5**
